# From Liquid to Solid-State Lithium Metal Batteries: Fundamental Issues and Recent Developments

**DOI:** 10.1007/s40820-023-01234-y

**Published:** 2023-11-20

**Authors:** Zhao Zhang, Wei-Qiang Han

**Affiliations:** School of Materials Science and Engineering, Zhejiang University, Hangzhou, 310027 People’s Republic of China

**Keywords:** Lithium metal batteries, All-solid-state lithium metal battery, Li dendrite, Solid electrolyte, Interface

## Abstract

The widespread adoption of lithium-ion batteries has been driven by the proliferation of portable electronic devices and electric vehicles, which have increasingly stringent energy density requirements. Lithium metal batteries (LMBs), with their ultralow reduction potential and high theoretical capacity, are widely regarded as the most promising technical pathway for achieving high energy density batteries. In this review, we provide a comprehensive overview of fundamental issues related to high reactivity and migrated interfaces in LMBs. Furthermore, we propose improved strategies involving interface engineering, 3D current collector design, electrolyte optimization, separator modification, application of alloyed anodes, and external field regulation to address these challenges. The utilization of solid-state electrolytes can significantly enhance the safety of LMBs and represents the only viable approach for advancing them. This review also encompasses the variation in fundamental issues and design strategies for the transition from liquid to solid electrolytes. Particularly noteworthy is that the introduction of SSEs will exacerbate differences in electrochemical and mechanical properties at the interface, leading to increased interface inhomogeneity—a critical factor contributing to failure in all-solid-state lithium metal batteries. Based on recent research works, this perspective highlights the current status of research on developing high-performance LMBs.

## Highlights

- The pursuit of high specific energy and high safety has promoted the transformation of lithium metal batteries from liquid to solid-state systems.
- In addition to high reactivity and mobile interface, all-solid-state lithium metal batteries (ASSLMBs) still faces severe inhomogeneity in mechanical and electrochemical properties.
- The inherent trade-off in ASSLMBs lies between ionic conductivity and electrochemical window, mechanical strength and interface contact adequacy.

## Graphical abstract

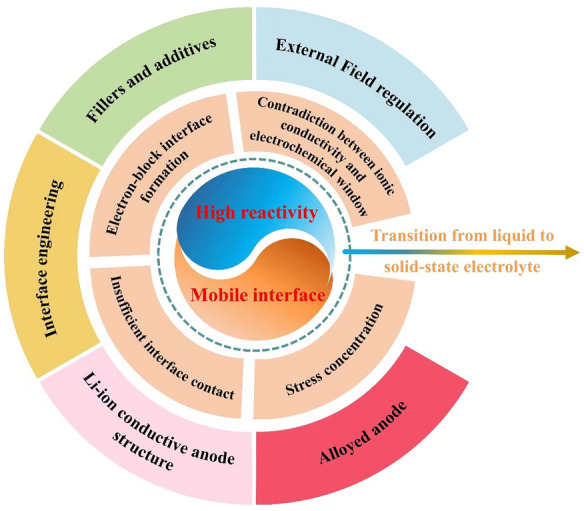

## Introduction

Since by Sony’s initial commercialization in the 1990s [1], lithium-ion batteries (LIBs) have progressively become omnipresent in modern life, finding extensive application in mobile phones, laptops, drones and other portable electronic devices [2, 3]. With the advent of large-scale manufacturing and significant cost reduction in LIBs, they are increasingly being employed in energy storage and conversion systems for renewable energy as well as electric and hybrid vehicles. This contributes to a reduction in carbon dioxide emissions into the atmosphere and a smarter life asking people less driving experience [4]. LIBs offer specific energy and energy density exceeding 270 Wh kg^−1^/650 Wh L^−1^, which are continuously improved through advancements in materials and production techniques [5]. However, the electric vehicle market demands an even higher energy density of over 400 Wh kg^−1^ to support a range of more than 1000 km [6]. Due to the limited capacity of graphite anode (372 mAh g^−1^), traditional LIB has approached its theoretical limits, hence there is a growing interest in next-generation batteries such as Li-air, Li-sulfur, and lithium metal batteries (LMBs) [7]. Among them, lithium metal anode (LMA) plays a crucial role due to its exceptionally high energy density (3860 mAh g^−1^), lowest reduction potential (− 3.04 V vs. standard hydrogen electrode) and low density (0.534 g cm^−3^) [8, 9]. Consequently, when coupled with typical intercalation-type cathode LiNi_0.8_Co_0.1_Mn_0.1_, lithium metal batteries can achieve gravimetric energy density of 575 Wh kg^−1^ and volumetric energy density 1414 Wh L^−1^ in a pouch cell [10]. Recently, this record has been surpassed by fully utilizing lithium-rich manganese-based cathode along with thin LMA, which results in rechargeable lithium batteries reaching an impressive gravimetric energy density of 711 Wh kg^−1^ and  a volumetric energy density of 1653 Wh L^−1^ [11].

Nevertheless, the commercialization of LMBs still faces a long and arduous journey [12]. The lowest electrochemical potential is a double-edged sword, as it not only enables a higher output voltage but also exacerbates the side reactions between LMA and electrolytes. In addition to infinite volume change and continuous fluctuation of internal stress, achieving stable passivation of LMA is challenging, which can lead to active Li consumption and subsequent impact on Li deposition behavior. The weak Li–Li bond renders metallic Li more susceptible to a dendritic electrodeposition [13], easily causing short circuit and pose severe safety hazards. To overcome these challenges, it is crucial to regulate Li deposition and prevent Li dendrites penetration [14, 15]. Over the past decade, considerable efforts have been dedicated to reviving such “Holy Grail” throughout the entire process of lithium electrodeposition, encompassing separator design, electrolyte formulation, solid-electrolyte interphase (SEI) layer engineering, and anode structure optimization. Extensive researches have also deepened our understanding of the structure and formation mechanism of SEI, as well as Li deposition behaviors. As a result, researchers now possess an array of strategies to harness the utilization of LMAs.

Unfortunately, the utilization of high energy–density anodes will inevitably lead to increased safety risks, underscoring the significance of substituting flammable and volatile liquid electrolytes with solid-state electrolytes (SSEs). Additionally, SSEs are anticipated to address Li dendrite penetration due to their exceptional mechanical strength [16]. Furthermore, the possibility of bipolar stacking can further augment energy and power density while reducing packaging requirements and circuit resistance [17]. Moreover, SSEs effectively mitigate electrode cross-talk, thereby preventing the undesired chemical interaction of dissolved active materials to eliminate the  primary source of instability for LMBs [18, 19]. Therefore, this envisions a roadmap for the transitioning from liquid-state to solid-state electrolytes [20]. However, there still exists a substantial gap between the practical application of all solid-state lithium metal batteries (ASSLMBs) and their theoretical potential due to the conflicting relationship between ionic conductivity and electrochemical window, as well as the delicate balance required for mechanical strength and interface contact, inherent surface or grain boundary defects [21–23]. In solid-state batteries, enhancing the compatibility between LMA and electrolyte shares many similarities with liquid-state systems; however, it also presents new challenges that inspire researchers’ pursuit of academic excellence.

This review summarizes the fundamental issues encountered by LMBs through a comparative evaluation of liquid versus solid electrolyte systems. In addition to the inherent trade-offs within SSEs concerning ionic conductivity and electrochemical window, as well as mechanical strength and interface contact, we particularly emphasize the need for clear elucidation of the impact arising from non-fluidity and sluggish dynamics in solid electrolytes. The key disparity between solid and liquid electrolyte systems lies in their heterogeneous mechanics and electrochemical properties at interfaces. Furthermore, we highlight similarities in improvement strategies during the transition from liquid to solid-state electrolytes, encompassing various aspects of battery architecture such as in situ and ex situ preparation techniques, adjustments related to external field factors, among others; their further evolution is also discussed.

## Fundamental Issues of LMA

**Fig. 1:**
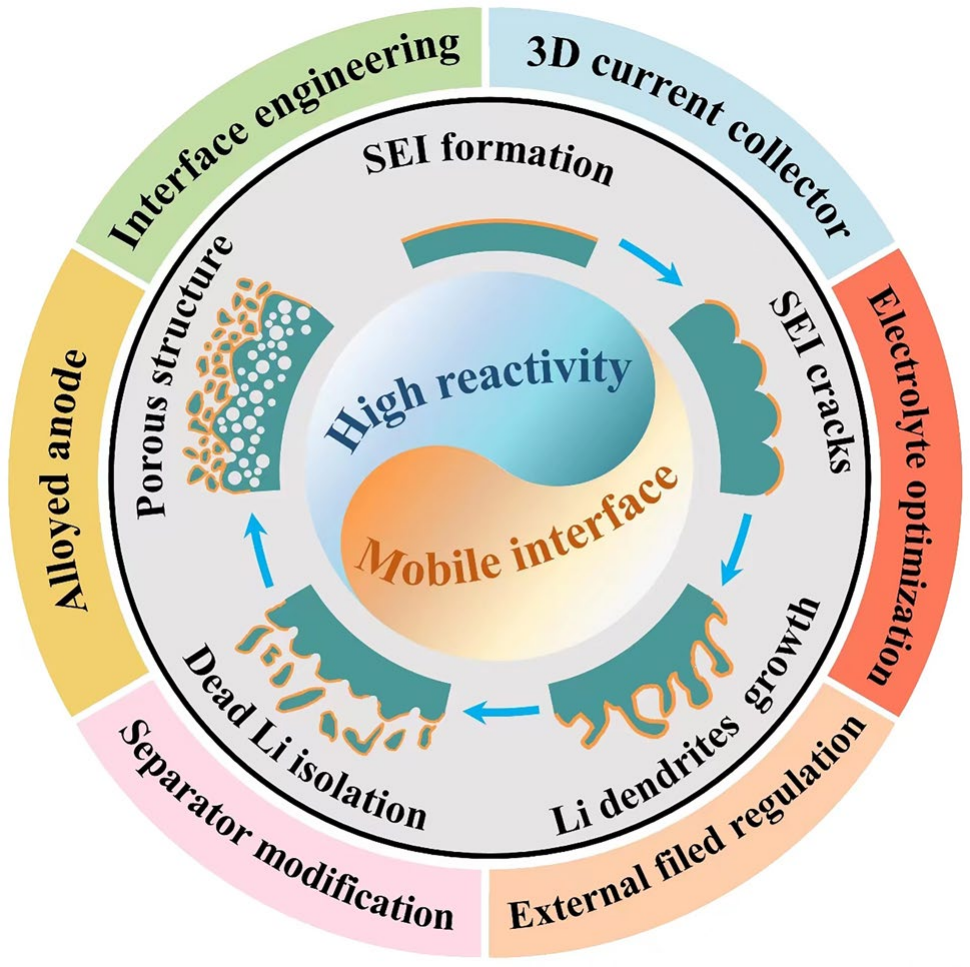
Schematic illustration of fundamental issues and corresponding strategies for lithium metal batteries (LMBs)

Despite the unparalleled advantages in energy density over conventional graphite anodes, practical application of LMAs still faces significant obstacles due to two primary reasons. Firstly, unlike the stable SEI formed during the initial cycles for graphite and silicon anodes, immediate passivation of the lithium metal surface occurs once upon contact with electrolytes and is continuously strengthened in subsequent cycling due to its high reactivity [24, 25]. Secondly, the electrochemical process involves distinct regions where independent oxidation or reduction reactions occurs, resulting in reactant dissolution and deposition. This migration of the reaction interface poses a fundamental challenge for LMBs. As a consequence of these combined electrochemical and mechanical actions mentioned above, Fig. 1 summarizes the evolution of surface morphology for LMAs. During Li plating/stripping processes, drastic changes in morphology occur leading to frequent SEI rupture that exacerbates interfacial side reactions, continuously consumes electrolyte, and promotes Li dendrite propagation from cracks. Additionally, uneven stripping from kinks or roots of Li dendrites can generate “dead” Li which becomes electrically isolated from substrates thereby significantly reducing coulombic efficiency [26]. After undergoing continuous cycling, the iterative process ultimately yields a porous Li anode structure characterized by volumetric expansion, thick SEI accumulation, and excessive dead Li, hindering the transport of Li-ions and contribute to capacity degradation. Furthermore, the conjunction of chemical side reactions and short circuits induced by Li dendrites poses a significant concern in mitigating safety hazards [27]. It should be noted that the ultra-high chemical reactivity and migration interface in nonaqueous electrolytes present fundamental challenges for LMBs distinct from those encountered in commercial LIBs.

### High Reactivity and SEI Layer

**Fig. 2:**
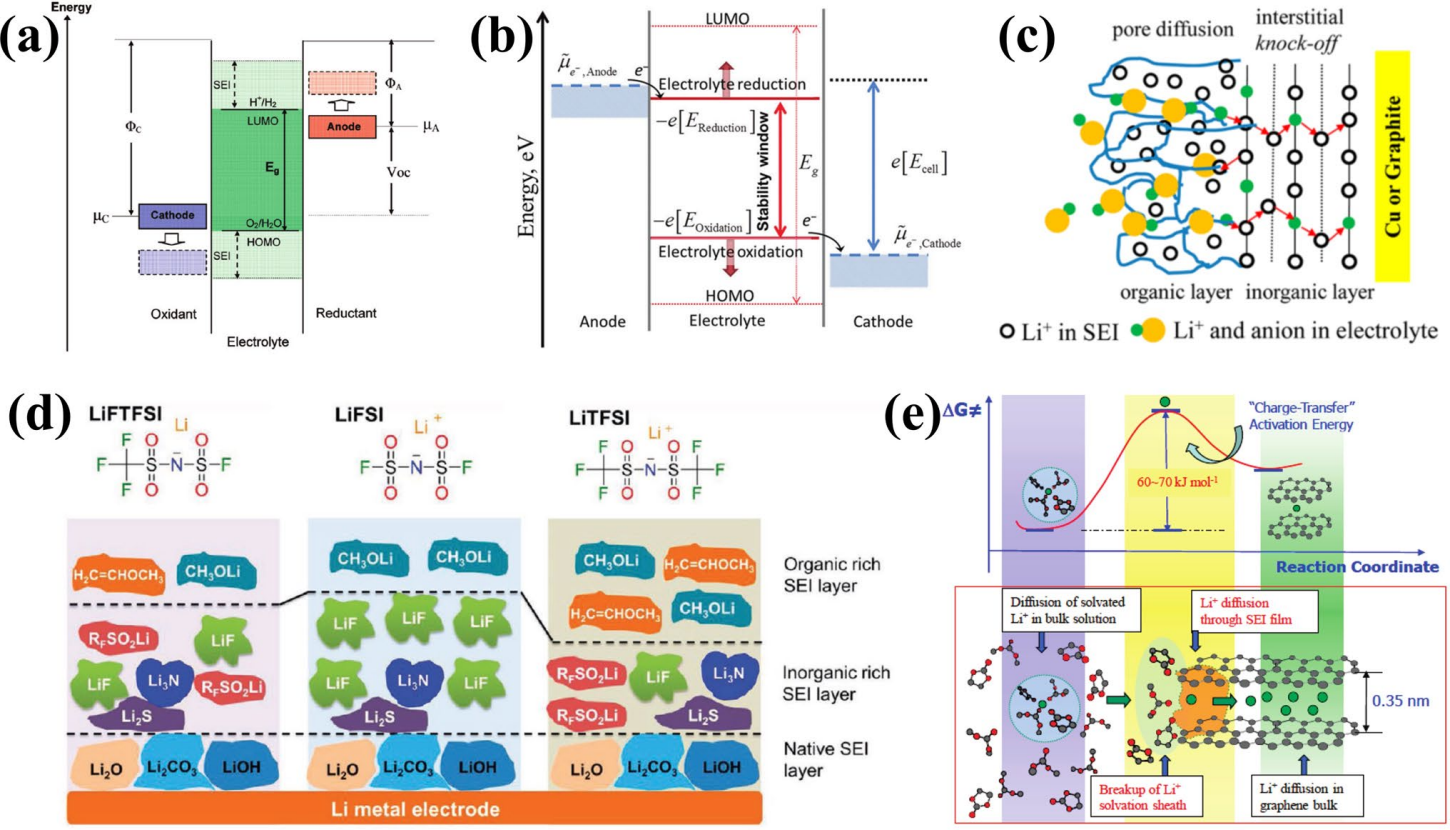
Schematics of **a** SEI formation mechanism and **b** revised mechanism in nonaqueous electrolytes. Reproduced with permission from [29] Copyright 2010, American Chemical Society. Reproduced with permission [31] Copyright 2018, Royal Society of Chemistry. **c** Schematics of double-layer structure of SEI and corresponding Li^+^ transport mechanism. Reproduced with permission from [36] Copyright 2012, American Chemical Society. **d** Schematic diagram of SEI layer formed on LMAs in three Li salts. Reproduced with permission from [41] Copyright 2018, American Chemical Society. **e** Schematic diagram of Li^+^ transport process and energy profile for Li^+^ desolvation. Reproduced with permission from [43] Copyright 2010, American Chemical Society

The high reactivity of LMA promotes the formation of a passivation layer, known as SEI, between it and the electrolyte. This layer is essential for facilitating the transport of lithium ions at the interface and regulating Li deposition behavior. Despite being intensively studied for over 40 years, it is still considered as “the most important but the least understood” aspect in lithium batteries due to its controversial formation mechanism and complex composition [28]. According to Goodenough et al.’s concept [29], it is widely acknowledged that the formation of a passivated SEI layer is closely related to the energy difference between the Fermi level of electrodes and the lowest unoccupied molecular orbital (LUMO)/highest occupied molecular orbital (HOMO) of electrolyte (corresponding energy levels are denoted as E_LUMO_ and E_HOMO_, respectively). As depicted in Fig. 2a, if the Fermi energy level of the anode (μ_A_) is higher than E_LUMO_, electrons are inclined to transfer from anode to electrolyte, provoking intrinsic reduction of electrolyte. Similarly, if the Fermi energy level of the cathode (μ_C_) falls below E_HOMO_, oxidation occurs at cathode-electrolyte interface. Only when both μ_A_ and μ_C_ fall within the electrochemical window of the electrolyte (defined as E_g_ =|E_LUMO _− E_HOMO_|), can SEI layer be prevented from forming at interface [30]. Peljo et al. considering complexity in electrolytes further revised this theory suggesting that Gibbs free energy difference of the reactants and products determines the redox potentials [31]. This is because HOMO and LUMO are derived from the electronic properties of isolated molecules through approximated electronic structure theory, which fails to capture the unique circumstances of interfacial redox reactions. Thus, it is recommended to substitute HOMO or LUMO energy levels with the potential for electrolyte reduction at negative potentials or the potential for electrolyte oxidation at positive potentials (Fig. 2b).

Due to the high reactivity of LMA, multiple reduction reactions occur simultaneously, resulting in the formation of a double-layered and mosaic-like SEI structure [32]. The mosaic SEI indicates that diverse products are precipitated and heterogeneously distributed within the SEI, facilitating Li^+^ transport across grain boundaries and amorphas regions [33, 34]. In terms of thickness direction, SEI layer features a double-layer structure [35]. The dense inorganic layer, located close to lithium metal surface, primarily consists of low oxidation species such as Li_2_O, LiOH, Li_2_CO_3_, LiF and Li_3_N. The porous outer layer is comprised of organic high oxidation species like ROLi, ROCO_2_Li and ROCOO_2_Li. Therefore, the transport mechanism for Li-ion undergoes a transition from pore diffusion in the porous organic layer solvated by solvent molecules and anions to knock-off diffusion in the dense inorganic layer of SEI [36, 37], as illustrated in Fig. 2c. And the resulting rate of Li^+^ diffusion significantly affects the electrodeposition behavior of metallic Li. Zhang’s research team presents a diffusion–reaction competition mechanism to elucidate the underlying reasons for different Li deposition morphologies [38]. Their findings demonstrate that a reaction-controlled process is more conducive to dendrite-free LMA rather than a diffusion-controlled one. The experiment measuring both the diffusion coefficient of Li^+^ ions and exchange current density using microelectrode also reveals a strong correlation between the rate of Li^+^ diffusion and deposited Li morphology [39].

The chemical composition of the SEI, particularly the inner inorganic layer, varies depending on the lithium salts employed [40]. Figure 2d illustrates the chemical composition of SEI in Li–S batteries under three distinct Li salts, where the SEI and electrochemical performance are influenced by the synergistic effect between lithium salt anions and polysulfide species [41]. Notably, different chemical composition exhibits varying energy barriers to ionic migration and electronic conduction. Therefore, it is crucial to establish a stable SEI with selectively favorable composition, such as Li_2_O, LiF and Li_3_N [25, 42]. Prior to transport through SEI, the solvated Li^+^ in bulk electrolyte must shed their solvation sheath, which is regarded as the rate-determining step for Li deposition (Fig. 2e), with an activation energy of approximately 60 ~ 70 kJ mol^−1^ [43]. In contrast, bare Li experiences an energy barrier of only about 20 kJ mol^−1^ when traversing through the SEI. Xu et al. further emphasize that the structure of solvated Li ion’s solvation sheath plays a central role in defining surface chemistry at the anode; solvent molecules or anions preferentially recruited into this inner solvation sheath would be preferentially reduced [44]. This provides a theoretical foundation for the artificial regulation of SEI via the optimization of the electrolytes.

### Mobile Interface and Li Deposition

**Fig. 3:**
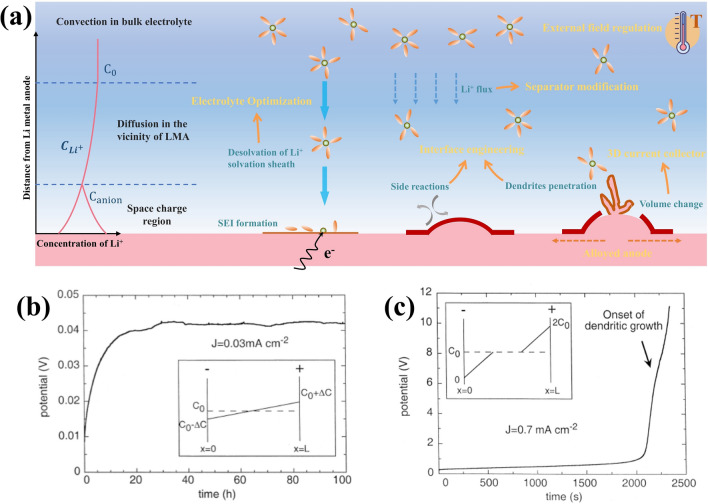
**a** Schematic illustration of Li-ion mass-transfer process with concentration gradient profiles and strategies affiliated by corresponding mechanism near the surface of lithium metal anode. The Li-ion concentration and potential profiles under two conditions: **b** < and **c** < when Li-ion depletion occurs. Reproduced with permission from [46] Copyright 1999, Elsevier. $dC/dx$ < $2{C}_{0}/L$ and **c** $dC/dx$ < $2{C}_{0}/L$ when Li-ion depletion occurs [46]

The independent reduction and oxidation process leads to the electrodeposition and stripping of metallic Li, which serve as the primary driving force for interface migration. The tendency of metallic Li to dendritic growth therein is one of the key issues. On one hand, this can be attributed to the intrinsic properties of lithium metal, namely its weak Li–Li bond and the high surface energy [13, 45]. On the other hand, the concentration gradient of Li^+^ in the electrolyte and non-uniform distribution of electric fields on the anode surface stimulate the propagation of Li dendrites. According to the Sand’s time model proposed by Brissot et al. and Chazalviel et al., the concentration gradient of Li-ion near the LMA electrode follows the Fick’s first law and the law of charge conservation under the influence of current density $\left( J \right)$ [46]:

1 $$\frac{\partial C}{{\partial x}}\left( x \right) = - \frac{J}{{eD\left( {1 + \frac{{\mu_{{Li^{ + } }} }}{{\mu_{a} }}} \right)}}$$

where $\frac{\partial C}{\partial x}\left(x\right)$ represents the Li-ion concentration gradient along the vertical direction of LMA as shown in Fig. 3a, $e$ is electronic charge, $D$ is the diffusion coefficient, ${\mu }_{a}$ and ${\mu }_{{Li}^{+}}$ are Li-ion and anionic mobilities. It can be inferred that the Li^+^ concentration gradient in the electrolyte is governed by the current density $J$. Assuming L as the distance between inner two electrodes and C_0_ as the initial Li^+^ concentration, two conditions can be predicted:

1. If $dC/dx$ < $2{C}_{0}/L$, The ion concentration distribution reaches a steady state with a constant gradient, and the potential approaches a constant value as depicted in Fig. 3b.
2. If $dC/dx$ > $2{C}_{0}/L$, there must exist a certain time (τ) when Li-ion concentration is completely depleted in the vicinity of LMA, which is called the “Sand’s time” [47], that causes local space charge region associated with large electric filed and triggers the rapid growth of Li dendrites [48] (Fig. 3c). The onset of Li dendrites under this unstable situation can be predicted by the “Sand’s time”.

2 $$\tau = \pi D\left( {\frac{{eC_{0} }}{{2Jt_{a} }}} \right)^{2}$$

3 $$t_{a} = \frac{{\mu_{a} }}{{\mu_{{Li^{ + } }} + \mu_{a} }}$$

where $t_{a}$ is anionic transfer number. Therefore, it can effectively delay the occurrence of Li-ion depletion and mitigate dendrites growth by moderately increasing the concentration of lithium ion in bulk electrolyte (C_0_), reducing the operating current density ($J$) and increasing the Li^+^ transference number ($t_{a}$).

In addition, considering the circumstance $dC/dx$ = $2{C}_{0}/L$, the critical current density value ${J}^{*}$, that is the maximum endurable current density in avoid of Li-ion depletion, is given by:

4 $$J^{*} = \frac{{2eC_{0} D}}{{t_{a} L}}$$

To sum up, the identification of Li dendrite triggers within cells is feasible by establishing a correlation between $J$ and ${J}^{*}$. Enhancing the ${J}^{*}$ of LMBs has proven to be an essential strategy in mitigating Li dendrite propagation [49]. However, it should be noted that low current densities may not eliminate lithium dendrites in practical applications due to the continuous promotion of gradual growth facilitated by ion concentration gradients in the electrolyte. Conversely, high current density does not necessarily result in more severe dendrite growth or a significant decline in electrochemical performance, as its impact on environmental factors must also be considered [50]. In reality, the behavior of Li deposition and subsequent migration at reaction interfaces is an intricate process influenced by multiple factors such as nucleation site, SEI layers and temperature [51–53].

## Design Strategies for LMB

Based on the fundamental issues posed by LMAs, including their high reactivity and mobile interface, as well as the formation mechanism of SEI and concreate Li deposition processes, the design strategies can be classified into the following six main aspects (Fig. 3a): interface engineering, 3D current collector, electrolyte optimization, separator modification, alloyed anodes, and external field regulation.

### Interface Engineering

Although the spontaneously generated SEI layer on LMAs can mitigate continuous interfacial side reactions, it always lacks sufficient mechanical strength to withstand SEI cracks during Li plating/stripping and facilitate fast Li-ion transport for homogeneous Li deposition. Therefore, it is crucial to regulate the composition and properties of SEI, and address any deficiencies through artificial interface engineering. In general, an ideal SEI layer should possess the following desirable characteristics [54]: (i) excellent physical and chemical stability to impede dissolution or reaction in electrolytes while maintaining structural and compositional integrity throughout cycling; (ii) superior electronic insulation to prevent the continuous Li consumption and electrolyte decomposition; (iii) high ionic conductivity to enable swift and homogeneous Li-ion transport across the SEI; (iv) exceptional mechanical strength and flexibility to suppress Li dendrites growth and accommodate volume changes during repeated cycles.

#### Membrane Coating

**Fig. 4:**
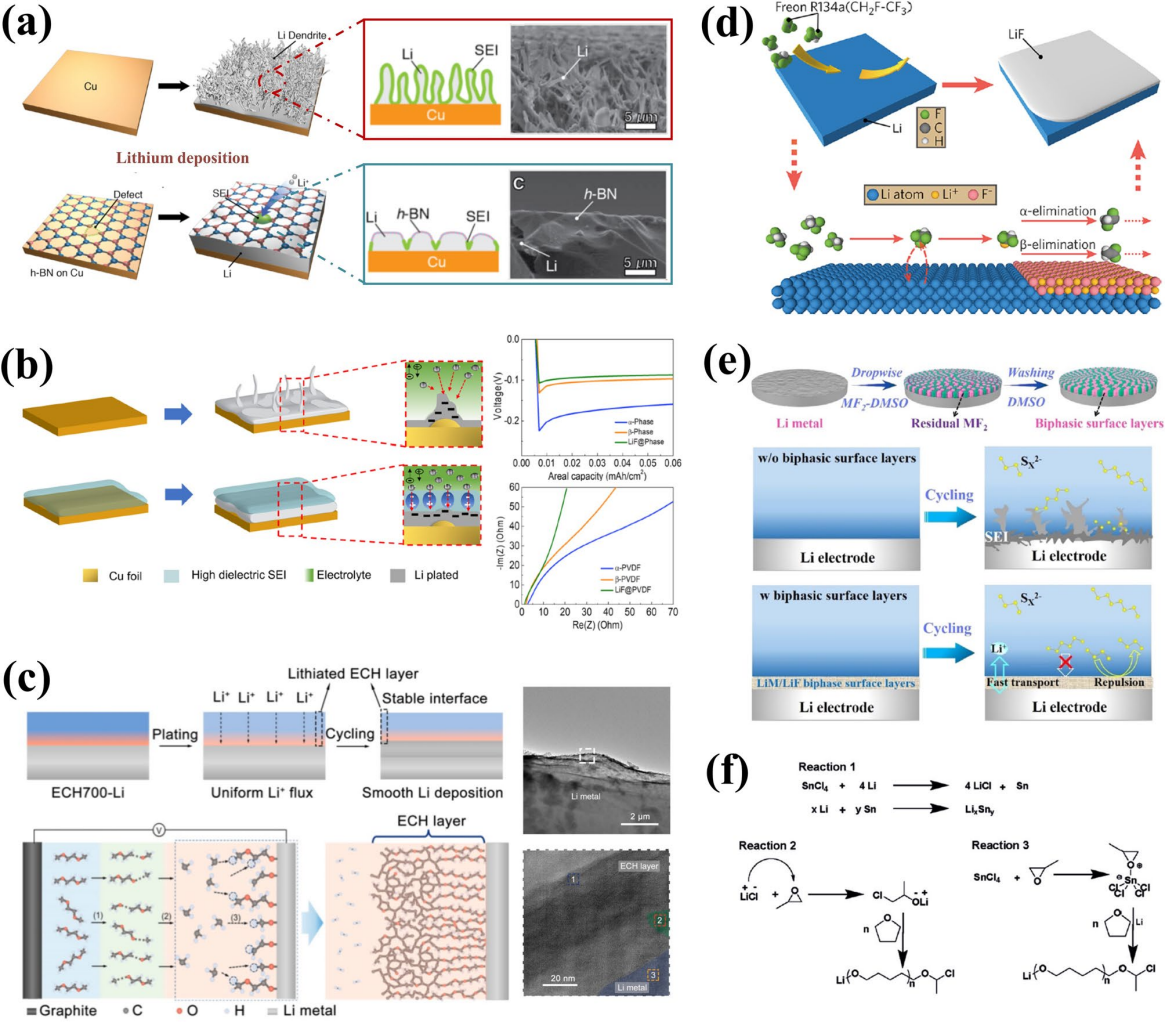
Interface engineering for LMAs in nonaqueous electrolytes. **a** Schematic diagrams of direct membrane coating by two-dimensional atomic crystal layers composed of hexagonal boron nitride (h-BN) and graphene. Reproduced with permission from [62] Copyright 2014, American Chemical Society. **b** Schematic illustration of mitigation mechanism of high-dielectric-constant artificial SEI and corresponding electrochemical performance. Reproduced with permission from [75] Copyright 2021, American Chemical Society. **c** Schematic diagrams of the construction of the carbon-based hybrid (ECH) interface on LMA surface via electrochemical pretreatment and corresponding TEM characterizations. Reproduced with permission from [81] Copyright 2022, American Chemical Society. **d** Schematic diagrams of surface LiF coating layer through in-situ chemical reactions. Reproduced with permission from [84] Copyright 2017, American Chemical Society. **e** Schematic illustration of the fabrication process of the robust biphasic surface layer (BSLs) and its inhibition in shuttling of Li–S batteries. Reproduced with permission from [85] Copyright 2021, Wiley–VCH. **f** Reaction mechanism for the formation of the PTMEG–Li/Sn alloy hybrid layer. Reproduced with permission from [93] Copyright 2019, Wiley–VCH.

Through physical methods such as physical vapor deposition (PVD), chemical vapor deposition (CVD), atomic layer deposition (ALD), doctor-blade coating, and spin-coating, a coated membrane with a thickness ranging from 5 nm to 10 μm can be directly fabricated on the surface of LMAs [55, 56]. The materials utilized include Al_2_O_3_ [57] and BN [58] with high Young’s modulus, carbon materials, lithium halides [59] and Li_3_N [60] for facilitating homogeneous Li deposition, as well as compliant polymers. The uniform and dense Al_2_O_3_ coating layer prepared by ALD deposition not only inhibits the side reaction at the interface, but also enhances the wettability of the Li surface towards organic electrolytes [61]. Similarly, the two-dimensional atomic crystal layers composed of hexagonal boron nitride (h-BN) and graphene offer exceptional interfacial protection for lithium anodes due to their high chemical stability and mechanical strength [62]. The propagation of Li dendrites is effectively suppressed beneath the stiff h-BN protective layer (Fig. 4a). The presence of point and line defects in these 2D layers allows for Li-ion penetration, leading to stable cycling with high coulombic efficiency even under high current densities. Zheng et al. [63] reported an interconnected, hollow amorphous carbon nanosphere coating that acts as a barrier between lithium metal and electrolytes. This amorphous nanosphere layer possesses high Young’s modulus of up to 200 GPa. It should be noted that the weak binding to the current collector allows for vertical movement, regulating the availability of empty space during the cycling, ensuring continuous contact with lithium anode and timely release of internal stress. The protective layer consists of dense fullerene (C_60_) can further provide excellent environmental stability that satisfies the pressing demand for the assembly of LMBs in air [64].

As a fast Li-ion conduction material, lithium nitride (Li_3_N) exhibits a remarkable ionic conductivity of 6 × 10^−3^ S cm^−1^ at room temperature and possess exceptional thermodynamic stability towards metallic Li. Consequently, it is considered as an essential component of the ideal SEI layer [65, 66]. Baloch et al. investigated the feasibility of directly applying a Li_3_N coating on the LMA to enhance Li deposition morphology and found that this coating layer could effectively reduce parasitic reactions at the interface, resulting in smooth surface morphology [60]. Furthermore, by magnetron sputtering a 150 nm-thick layer of LiF, significant alterations in the deposition behavior of Li were observed along with effective prevention of dendrite propagation [67]. These beneficial effects can be primarily attributed to several key characteristics exhibited by LiF: (i) wide electrochemical window (up to 6.4 V vs. Li/Li^+^) [68]; (ii) Low solubility to ensure physical stability in nonaqueous electrolytes; (iii) extremely low electron conductivity (10^−13^–10^−14^ S cm^−1^) to block electron tunneling [25]; (iv) high surface diffusion coefficient and surface energy. These outstanding properties possessed by LiF make it an indispensable constituent within the optimal SEI structure. Despite the sluggish bulk Li-ion transport in LiF, it plays a crucial role in regulating the homogeneous deposition of Li^+^ flux. Other inorganic coatings, such as amorphous Li_3_PO_4_ film (with a low electron conductivity of 1.4 × 10^−10^ S cm^−1^), have also been proven effective for inhibiting growth of dendritic lithium and extending lifespan of LMBs.

Polymer films are excellent candidates for interface engineering in LMAs due to their inherent flexibility, enabling them to effectively accommodate volume and stress fluctuations during Li plating/stripping processes. Various polymers such as polyethylene oxide (PEO) [69], poly(vinylidene difluoride) (PVDF) [70], polyacrylonitrile (PAN) [71], perfluoropolyether [72], and polyvinyl alcohol (PVA) [73], have been successfully employed to enhance the electrochemical performance and stability of LMAs. Additionally, there is a growing trend towards utilizing organic–inorganic composite layer that combine the advantages of high modulus inorganic nanoparticles with flexible polymers. For instance, Jang et al. proposed a hybrid protective layer composed of zirconia (ZrO_2_) particles and poly(vinylidene fluoride-co-hexafluoropropylene) (PVDF-HFP), which not only facilitates fast Li^+^ transport at the interface but also suppresses Li dendrites propagation [74]. Furthermore, recent studies have increasingly focused on exploring the dielectric properties of artificial interface layers due to their impact on the redistribution effect of Li-ion flux near high-dielectric coating layers [75]. During the plating process, preferential deposition of Li occurs at protuberance where its further growth exacerbates the localized electric field and leads to surface roughening (Fig. 4b). The continuous consumption of Li-ions in the vicinity of protrusions accelerates the depletion of local Li^+^ concentration and triggers dendritic growth. While, a high-dielectric coating offers a dipole layer rearranged alongside the surface of lithium anode, which can guide uniform lithium-ion flux. Despite potential protrusion formation, the high-dielectric layer disperses lithium-ion pathways through its conformal charge-polarized layer, mitigating irregularities in local ion concentrations. The effective regulation of lithium-ion flux leads to homogenous Li deposition, resulting in the lower overpotential and high reversibility of LMAs. Enhancing the dielectric properties of synthetic coating layers can be achieved by replacing β-PVDF with materials having relatively higher dielectric constants or incorporating fillers such as LiF [76]. Chen’s team has developed a high dielectric interface layer composed of an aligned polymer matrix embedded with spatially confined LiF nanoparticles, which effectively homogenizes the electric field distribution near the surface of metallic Li anode[77]. Besides, the aligned pores with LiF nanoparticles can promote the Li-ion transport across the interface. Under the synergistic effect between highly polar β-phase PVDF and LiF nanoparticles, symmetric Li/Li cells exhibit exceptional cycling stability exceeding 900 h.

#### Electrochemical Pretreatment

The electrolyte plays a pivotal role in determining the structure and composition of SEI. Therefore, electrochemical pretreatment of LMAs in carefully selected electrolytes is a significant approach for fine-tuning SEI composition and electrochemical properties [78]. The advantage lies in its ability to facilitate arbitrary modifications to solvent or additive type and content, without being constrained by practical requirements such as electrolyte viscosity or long-term stability of cathode materials during cycling [79]. Notably, an exceptional implantable SEI can be generated through electrochemical pretreatment in LiTFSI (1 M)-LiNO_3_ (5 wt%)-Li_2_S_5_ (0.02 M)-DOL/DME ternary salt electrolyte, with a polycrystalline structure that consists of Li_3_N, Li_2_O, LiF, Li_2_S and Li_2_SO_4_ [80]. Importantly, LMAs with this implanted SEI demonstrate excellent compatibility with sulfur and LiNi_0.5_Co_0.2_Mn_0.3_O_2_ (NCM) cathodes, even in the ester electrolyte composed of LiPF6 (1 M)-EC/DEC. The research group led by Tao successfully fabricated a stable nanostructured electrolytic carbon-based hybrid (ECH) interface on the surface of LMA through the electrolysis of 1,2-dimethoxyethane (DME) at an ultrahigh voltage of 700 V (Fig. 4c) [81]. The resulting ECH layer consists of an inner phase dominated by lithium oxide and polymer layers, as well as an outer amorphous carbon layer. This effectively seals the LMA surface, preventing undesired chemical reactions, physically blocking Li dendrite growth, and inhibiting Li pulverization. Moreover, the ECH layer shows strong Li^+^ affinity that leads to the continuous Li^+^ adsorption and dendrite-free Li deposition.

#### In-situ Chemical Approach

Reactive gases or solvents can readily form an artificial interface layer on the surface of LMAs through in-situ chemical reactions, resulting in a uniform and compact SEI under the reactions with N_2_ [82], F_2_ [83], Freon [84] and other gases. Cui and colleagues [84] developed a conformal LiF coating on Li metal surface with commercial Freon R134a as the reagent (Fig. 4d). Furthermore, gaseous Freon can be applied to achieve a uniform LiF coating onto 3D layered Li-reduced graphene oxide (Li-rGO) anodes, which reduces side reactions and enhances cycling stability of lithium-metal batteries.

In most cases, the artificial SEI layer that enables intimate contact with LMAs is obtained through redox reactions between metallic Li and liquid reagents. For example, a robust biphasic surface layer (BSL) is in-situ formed by the simple substitution reaction between metallic Li and metal fluoride (like SnF_2_, InF_3_ and ZnF_2_) in the dimethyl sulfoxide (DMSO) solution (Fig. 4e) [85]. The resulting BSL layer obtained is composed of lithiophilic alloy (like Li-Sn, Li-In, and Li-Zn) and LiF phases on the LMA surface, effectively inhibiting the shuttle effect and dendritic growth while enhancing interface reaction kinetics for rapid Li^+^ transport. With the assistance of a DME-based CuF_2_ solution, Yan et al. produced an armored mixed conductive SEI with high elastic modulus (12.9 GPa) and rapid Li-ion transport, and verified its inhibition effect on Li dendrites through in-situ optical observation [86]. Similarly, interface engineering often involves metal halides, like SbF_3_ [87], InF_3_ [88], CuCl_2_ [89] and SnCl_4_ [90], which exhibit essential advantages in nano-scaled SEI construction and much smaller interface impedance compared with physically coated layers. A smart SEI layer with excellent binding ability and stability can be created via the in-situ reaction between lithium and polyacrylic acid (PAA) [91]. Due to the superior elasticity of the polymer matrix, the resulting LiPAA layer enables self-adaptive interface regulation to accommodate volume changes during dynamic Li plating/stripping processes.

Combining an elastic polymer matrix with reactive inorganic particles, Cui’s group developed an artificial interface layer composed of Cu_3_N nanoparticles embedded within styrene butadiene rubber (Cu_3_N + SBR) [92]. This material demonstrated high mechanical strength, outstanding flexibility, and superior Li-ion conductivity—all essential properties for achieving an ideal SEI. Importantly, upon contact with Cu_3_N nanoparticles, a spontaneous transition to Li_3_N occurred on the surface of the LMA, resulting in accelerated Li-ion transport and homogenized Li-ion flux. In the meantime, the flexible SBR effectively maintains structural integrity and mitigates Li pulverization during Li plating/stripping. Furthermore, Guo’s research group adeptly utilized the nucleophilic properties of SnCl_4_ reaction products to induce successful polymerization of tetrahydrofuran (THF) solvent, leading to the preparation of a hybrid layer consisting of poly(tetramethylene ether glycol) (PTMEG)-Li/Sn alloy [93]. The possible reaction mechanism for the ring-open polymerization is illustrated in Fig. 4f. The hybrid layer not only facilitates the rapid transmission of Li-ions due to ample Li vacancies in Li/Sn alloy, but also exhibits significant hydrophobicity owing to high concentration of alkyl groups in its structure, contributing to the excellent environmental stability of LMAs.

### 3D Current Collector

**Fig. 5:**
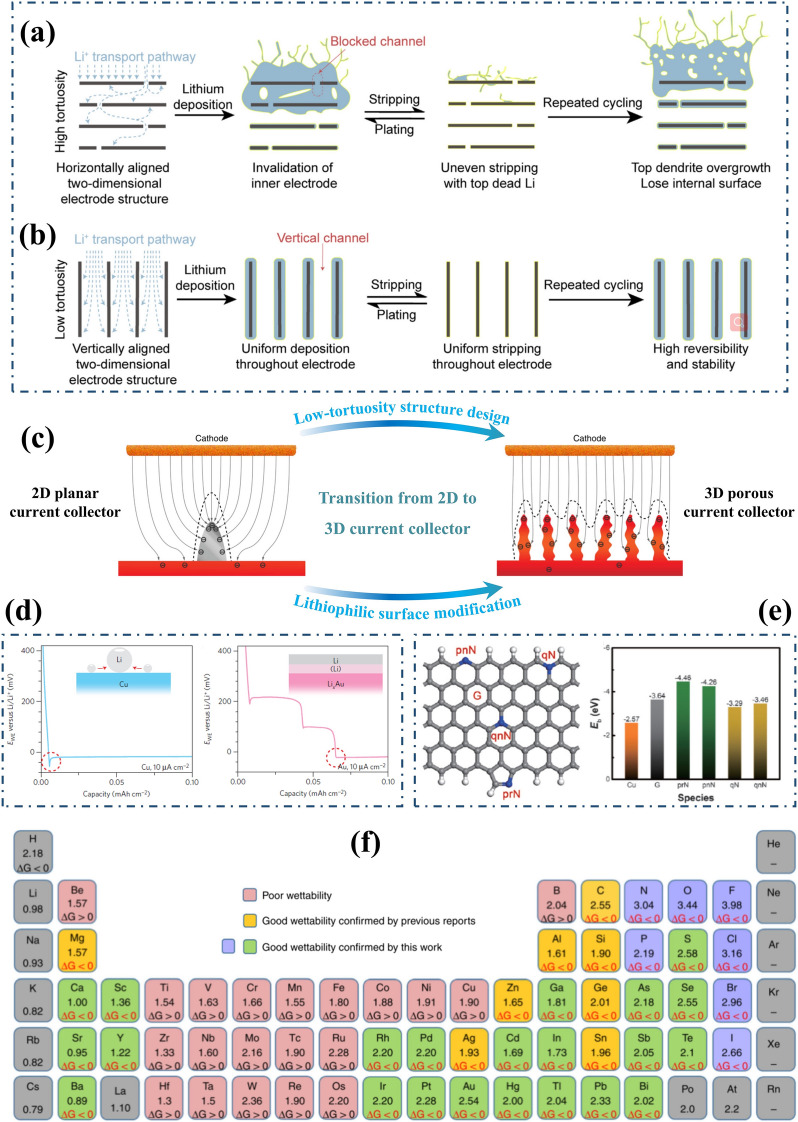
The construction of 3D current collector for LMAs. Schematic illustration of the effect of tortuosity on the structure evolution of LMAs during the cycling: **a** high tortuosity, **b** low tortuosity. Reproduced with permission from [111] Copyright 2020, Elsevier. **c** Schematic illustration of the transition from 2D planar to 3D current collector. Reproduced with permission from [96] Copyright 2015, The Authors, published by Springer Nature. **d** Overpotentials of galvanostatic Li deposition on Cu and Au substrate. Reproduced with permission from [117] Copyright 2016, The Authors, published by Springer Nature. **e** Schematic diagram and calculated binding energy of a Li atom with Cu, graphene, and different functional groups of N-doped graphene. Reproduced with permission from [121] Copyright 2017, Wiley–VCH. f Electronegativities of various elements in the periodic table and related Gibbs free energy () of elements or compounds reacted with molten Li. Reproduced with permission from [124] Copyright 2019, The Authors, published by Springer Nature

The transformation from a 2D planar to 3D current collector can significantly alleviate the volume and internal stress changes of LMAs during cycling, thereby addressing the recurring SEI cracks and continuous active Li/electrolyte consumption. According to Sand’s model [46, 94], it can effectively delay the growth of Li dendrites growth by reducing local current density, as sand’s time is inversely proportional to the operating current density [95]. In 2015, Guo et al. reported the synthesis of a jungle-like porous layer with copper fiber bundles as current collectors of LMAs, which was fabricated by the thermal reduction of Cu(OH)_2_ nanowires (Fig. 5c) [96]. On a planar current collector, metallic Li tends to deposit at the sharp edges of previously deposited Li particles where charges accumulate in the electric field. This phenomenon amplifies the growth of Li dendrites and further distorts the distribution of electric field. In contrast, the 3D Cu foil provides numerous protuberant tips on its submicron fibers that act as charge centers and nucleation sites due to its extended specific surface area that is 45 times higher than traditional Cu foils. The electric field is engineered to exhibit a nearly uniform distribution, ensuring the charges are evenly dispersed along the Cu skeleton. It is anticipated that Li will nucleate and grow on a submicron-sized Cu skeleton, effectively filling the pores of the 3D current collector and ultimately forming a relatively uniform Li surface. This approach significantly mitigates the risk of dendrite penetration and thermal runaway. Zhao et al. constructed a 3D Cu framework with uniform, smooth, and compact porous network by the electrochemical etching of Cu–Zn alloy [97]. The precisely engraved continuous structure endows high electric conductivity and satisfactory mechanical properties. The uniform pores with large internal surface area guarantee well-dispersed current density for homogeneous Li deposition and accommodation. And the smooth surface promotes the formation of a stable SEI layer, which effectively inhibits Li dendrite and dead Li accumulation. These findings highlight dealloying technique as an alternative method for obtaining metallic 3D current collectors [98]. In addition, other copper-based skeleton materials featuring microstructures, such as Cu mesh [99] and Cu foam [100, 101], present themselves as viable alternatives for 3D current collectors.

Frameworks possessing a 3D porous structure, excellent electronic conductivity, and electrochemical stability towards metallic Li are suitable for the development of 3D current collectors in LMBs [102]. In addition to metallic materials such as nickel (Ni) foam [103, 104], titanium (Ti) foam [105], and molybdenum (Mo) mesh [106], the much lighter carbon materials like carbon nanospheres, carbon nanotubes (CNTs), graphene, carbon fibers and carbon cloth have also garnered significant attention [107, 108]. Hollow carbon fibers are fabricated to confine Li deposition and prevent the unprecedented growth of Li dendrites by increasing nucleation sites due to their enlarged electrochemically active surface area [109]. What’s more, optimizing the porous anode structure is crucial for enhancing Li-ion transport in 3D current collector structural design. Zhang et al. proposed a self-supporting carbon membrane with vertically aligned micro-channels as the framework for LMA, to adapt to giant volume and internal stress changes during Li plating/stripping process [110]. This unique design offers homogenized, straight, and direct ion transport paths within the low-tortuosity carbon membrane obtained. As a result, it enables uniform and fast delivery of Li ions over long electrode distances as well as high-rate operation. As a result, the fabricated electrodes exhibit extraordinary cyclic stability under high current density up to 40 mA cm^−2^ and high areal loading up to 40 mAh cm^−2^ with the overpotential of only 30 mV. It should be noted that tortuosity plays a crucial role in characterizing the intricacy of Li-ion transportation in porous electrodes since it is influenced by the geometrical complexity of their microstructure [111]. Tortuosity directly affects actual liquid-phase diffusivity and electronic conductivity of these porous electrodes.

5 $$K_{eff} = \frac{K\varepsilon }{\tau }$$

6 $$D_{eff} = \frac{D\varepsilon }{\tau }$$

where ${K}_{eff}$ and $K$ represent effective and intrinsic electron conductivity, respectively; ${D}_{eff}$ and $D$ represent effective and intrinsic diffusivities in liquid-phase, respectively; $\epsilon$ is porosity. Above two Eqs. (5) and (6) serves as the definition of tortuosity ($\tau$). Figure 5a, b illustrates the evolution of Li anode structure with different tortuosity during Li plating/stripping process. As shown in Fig. 5a, in a high tortuosity anode structure with horizontally aligned layered conductive sheets, metallic Li inclines to be deposited and stripped only at the upper surface, due to longer ion transport pathways to the middle or bottom of the electrode. Over time, the accumulated Li on the top gradually obstructs Li-ion transport channels into the inner electrode, exacerbating excessive deposition on the surface. Even after stripping, residual dead Li and SEI lead to a decreased active surface area, intensifying local electric field and current density heterogeneity that further aggravates uneven Li accumulation. This outcome renders carefully designed multilevel electrode structures ineffective. In contrast, the vertically aligned structure, with ultra-low tortuosity provides straight, inward Li-ion transport pathways, enabling highly reversible uniform Li delivery (Fig. 5b), without excessive Li deposition at the top surface. In summary, conducting further structural improvements are essential for practical applications of LMBs, where tortuosity serves as one critical parameter for evaluating structural design.

From the perspective of metal electrodeposition, the process of Li deposition can be divided into two main steps [112]: (1) diffusion of Li-ions to the electrode substrate and their subsequent reduction to Li atoms; (2) migration of these atoms along the substrate until they enter the crystal lattice and undergo electro-crystallization. In general, mass-transfer through the SEI layer is usually determines the rate due to much faster charge-transfer kinetics for Li-ion reduction, as indicated by a large exchange current density [113]. Therefore, it remains crucial to artificially regulate the structure and composition of SEI in order to expedite the mass-transfer process of Li-ions at the interface during 3D current collector construction. Shen et al. reported uniform and vertical Cu_7_S_4_ nano-flake arrays on Cu substrate to form a Li_2_S-enriched SEI via their electrochemical reduction with metallic Li [114]. A higher content of Li_2_S in the SEI layer is more effective in homogenizing Li^+^ flux and inhibiting the formation of Li dendrites, resulting in reduced dead Li. This approach achieved a high level of coulombic efficiency, reaching 98.6%, over 400 cycles at 1 mA cm^−2^. Moreover, the chemical reaction of Cu_3_N nanowires results in an SEI layer rich in Li_3_N, which promotes ionic conductivity at the interface and enhances the reversibility of LMAs [115].

Due to the weak Li–Li bond and high surface energy, metallic Li is prone to form a one-dimensional dendrite structure during electro-plating process [13, 116]. By altering the surface energy state for Li deposition, to achieve a transition to heterogeneous nucleation is a crucial direction for the further development of 3D current collectors [108]. Cui’s group investigated the nucleation pattern of metallic Li on a list of 11 elemental substrate: Au, Ag, Zn, Mg, Al, Pt, Si, Sn, C, Cu and Ni. They discovered a substrate-dependent growth phenomenon that enables selective Li deposition [117]. The nucleation overpotential was defined as the difference between the voltage dip bottom and the flat part of the voltage plateau during galvanostatic Li deposition. As shown in Fig. 5d, the Cu or Ni substrates exhibit an overpotential of approximately 40 mV required to overcome the heterogeneous nucleation barrier caused by significant thermodynamic mismatch with metallic Li. On the other hand, Au, Ag, Zn and Mg substrate have a definite solubility in lithium without any obvious overpotential indicating negligible nucleation barrier. Guided by principles governing Li metal nucleation behavior, preferential deposition of Li on Au particles leads to selective deposition and stable encapsulation of metallic Li inside hollow carbon spheres, thereby eliminating dendrite formation and enabling improved cycling performance.

Except for metallic elements, non-metallic elements such as halogens and B, N, O, P, S, also possess strong bonding energies with Li-ions [118–120]. Zhang et al. investigated the binding energy of a Li atom with Cu, graphene, as well as different functional groups of N-doped graphene (Fig. 5e) [121, 122]. The lithiophilic pyrrolic N and pyridinic N demonstrate relatively larger binding energies of − 4.46 and − 4.26 eV compared to graphene (− 3.64 eV) and Cu (− 2.57 eV), thereby guiding the nucleation of metallic Li and resulting in uniformly deposited Li anodes. Li et al. fabricated a lithiophilic carbon cloth co-doped by nitrogen and phosphorous, which enables the uniform loading of molten Li and highly reversible Li stripping/plating [123]. Wang et al. furtherly calculated the Gibbs free energy changes (${\Delta }_{r}G$) of elements or compounds reacted with molten Li [124]. According to Fig. 5f, the elements in the periodic table can be divided into lithophilic elements (${\Delta }_{r}G<0$) and lithiophobic elements (${\Delta }_{r}G>0$), providing a theoretical basis for enhancing wettability and electrochemical performance on 3D current collectors. Niu et al. reported an amino-functionalized mesoporous carbon nanofiber cloth as the framework of LMA to fully utilize the strong interaction between –NH and Li [125]. The functional groups change the Li wettability of the carbon host from non-wetting to superwetting, and the pore channels or the cavities provide preferred initial nucleation sites for Li plating. As a result, Li is deposited uniformly along the carbon fibers to form stable SEI layer, without triggering Li dendrites growth. This successful combination of conductive frameworks and lithiophilic modifications enables the realization of a pouch cell with a high energy density of 350–380 Wh kg^−1^ and stable cycling life up to 200 cycles.

The 2D material MXene possesses satisfactory electronic conductivity, high surface area and abundant functional groups, making it a suitable candidate for constructing 3D current collector [126–128]. With its numerous preferred nucleation sites, MXenes as conductive skeleton can effectively disperse local current density and mitigate polarization during cycling [129, 130]. The emergence of novel low-dimensional materials significantly expands the possibilities for developing 3D current collectors and accelerates the progress of LMBs [131–133].

To summarize, the design of 3D current collectors is progressively advancing towards greater sophistication. This entails not only emphasizing the development of conductive microstructures but also considering modifications or coatings on lithiophilic nucleation sites. Furthermore, this underscores the practical significance of employing low-dimensional materials.

### Electrolyte Optimization

#### Electrolytes with Novel Additives

**Fig. 6:**
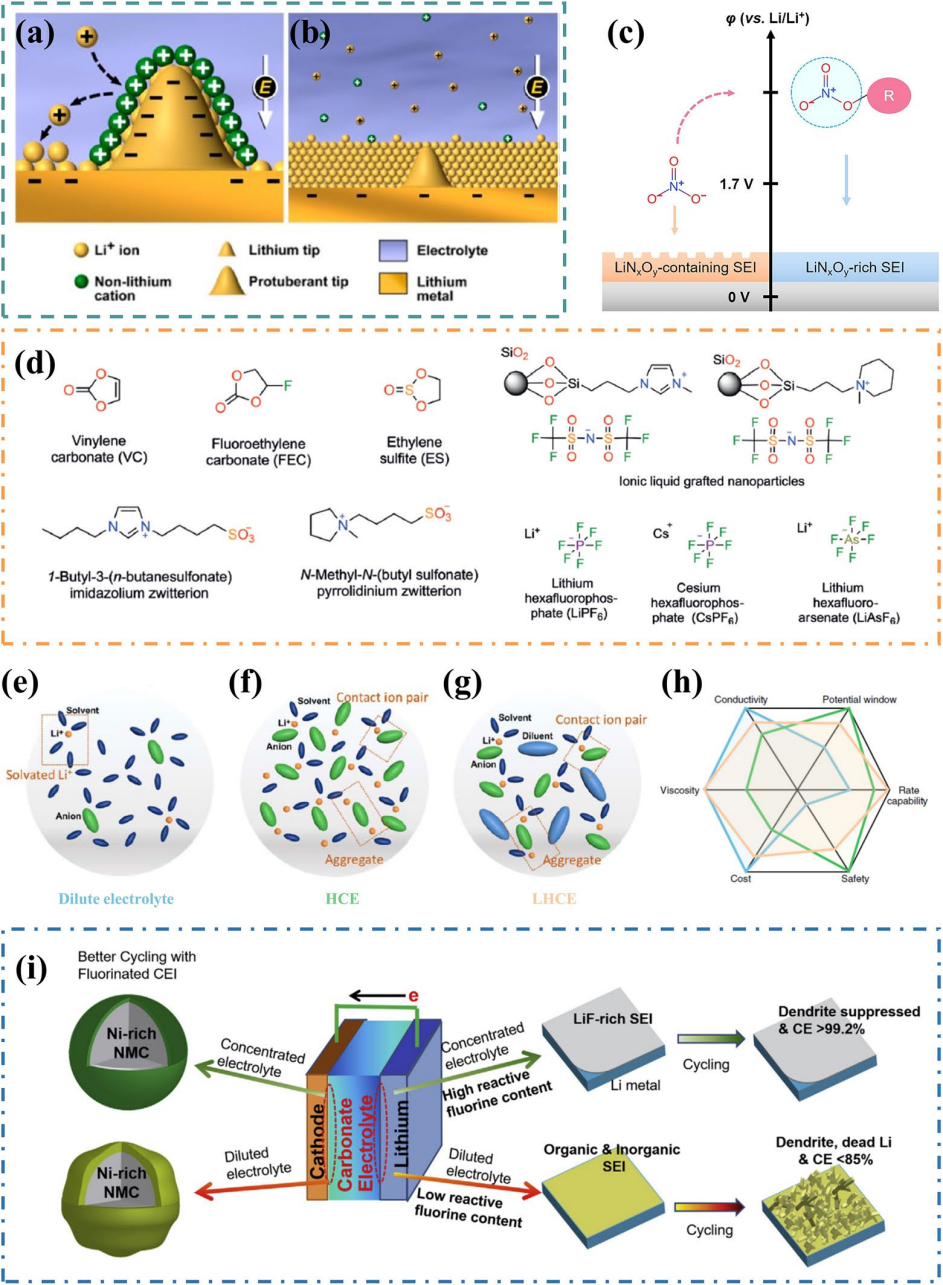
The electrolyte modification for LMAs. Illustration of Li deposition process based on self-healing electrostatic shield (SHES) mechanism: **a** without and **b** with 0.01 M CsPF6 addition. Reproduced with permission from [135] Copyright 2013, American Chemical Society. **c** Schematic illustration of a modification of NO3− in stabilizing the SEI. Reproduced with permission from [157] Copyright 2022, Wiley–VCH. **d** Chemical structures of additives used for passivating LMAs. Reproduced with permission from [140] Copyright 2018, Wiley–VCH. Electrolyte structures of e conventional dilute electrolyte, **f** high concentration electrolyte (HCE) and **g** localized high concentration electrolytes (LHCE). Reproduced with permission from [176] Copyright 2021, IOP Science. h Comparison of the properties and performances of dilute electrolyte, HCE and LHCE. Reproduced with permission from [182] Copyright 2019, The Authors, published by Springer Nature. **i** Schematic illustration of the effect of the reactive F-content in the carbonate-based HCE on LMA and Ni-rich cathode. Reproduced with permission from [178] Copyright 2018, Elsevier.

The formation of SEI layer and deposition behavior of Li are highly dependent on the composition and concentration of nonaqueous electrolytes. Therefore, even a small amount of additive (typically less than 5% by weight or by volume) can significantly impact the performance of LMBs. It is worth noting that trace amount of CsPF_6_ and RbPF_6_ can achieve dendrite-free LMA in carbonate electrolyte through a self-healing electrostatic shield (SHES) mechanism [134, 135]. The relevant mechanism is illustrated in Fig. 6a, b. At low concentrations, Cs^+^ and Rb^+^ cations practically exhibit a much lower reduction potential compared to the standard potential of Li-ions. During the process of lithium plating, these additive cations thermodynamically absorb onto the protuberances of deposited Li particles, forming a positively charged electrostatic shield around them. This force repels excessive Li^+^ away from the tip and guides their deposition on adjacent regions, resulting in a homogenous and compact nanorod-structured morphology (Fig. 6b). Additionally, incorporating insoluble solid nanoparticles can sometimes enhance the electrochemical performance in LMBs. Peng et al. [136] reported a novel solid additive, that is nano-scaled CaCO_3_ particles, for the stable cycling of LMA in commercial carbonate electrolyte. The working principle behind this method relies on sustained release: dispersed nano CaCO_3_ particles continuously absorb by-products generated during parasitic reactions and release Ca^2+^. This not only inhibits the tip growth of metallic Li through electrostatic shielding effects but also effectively promotes the formation of a stable F-rich SEI. Similarly, hieratically porous LiF nanoparticles were designed by Yao’s group to facilitate the formation of a highly fluorinated SEI layer for stabilizing LMA without involving any chemical reactions [137].

Most additives function sacrificially, possessing lower LUMO energy levels and preferentially reacting with metallic Li to form a compact and stable SEI layer during the initial period or in direct contact [138, 139]. Over the past decade, various types of additives and their combinations have been explored and utilized (Fig. 6d) [140]. These additives comprise solvents, such as vinyl carbonate (VC) [141], fluoroethylene carbonate (FEC) [142, 143], ethylene sulfite (ES) [144]; Li salts, including LiNO_3_ [145–147], LiPO_2_F_2_ [148], LiFSI [149]; and other materials like Sn(OTf)_2_ [150], I_2_ [151] and AlCl_3_ [152]. The active additives persist long-term in electrolytes, promptly restoring the SEI layer once fresh Li is exposed due to interface cracking, thereby maintaining stable electrochemical properties of LMAs [153]. Among them, LiNO_3_ has been widely adopted in various electrolytes, especially for Li–S batteries, where it is regarded as one of the most effective additives [154]. The relatively higher reduction potential of NO_3_^−^ (~ 1.7 V versus Li/Li^+^) leads to the preferential formation of LiN_x_O_y_ in SEI, furtherly promoting spherical nucleation and homogeneous Li deposition with high CE [155, 156]. Hou et al. connected NO_3_^−^ to an ether-based moiety in isosorbide dinitrate (ISDN) as an enhanced nitrate additive, disrupting the resonance structure and enhancing the reducibility of NO_3_^−^ (Fig. 6c) [157, 158]. The decomposition of NO_3_^−^ in ISDN enriches the SEI with abundant LiN_x_O_y_ spices, thereby imparting high-capacity retention to Li–S batteries under practical conditions. The effective utilization of LiNO_3_ in ester-based electrolytes for high-voltage batteries has recently garnered significant attention [159]. For instance, pre-embedding LiNO_3_ into the slurry containing Li powder and PVDF-HFP binder can act as a sustainable reservoir for controlled release into the electrolyte, facilitating SEI repair during cycling. Yan et al. discovered that a trace amount of CuF_2_ dissolution promoter could achieve up to 1 wt% solubility of LiNO_3_ in EC/DEC electrolyte [147]. This is attributed to Cu^2+^ possessing strong electron-withdrawing ability and altering the dissolution equilibrium of LiNO_3_. In the carbonate electrolyte, LiNO3 is reduced at 1.4 V, resulting in the formation of a stable LiNxOy/Li_3_N SEI layer that contributes to a high average coulombic efficiency (CE) of 99.5% when coupled with NCA cathodes. In addition, solvents with high donor number (DN), such as dimethyl sulfoxide (DMSO) [160], tetraglyme (TEGDME) [161], ethylene glycol diacetate [162], have been identified for use as dissolution cosolvent for LiNO_3_ to enhance the compatibility of LMAs and carbonate electrolytes.

FEC serves as a representative solvent for constructing a fluorinated SEI. On the surface of Li metal, a single HF molecule can be extracted from FEC to form a VC molecule, which subsequently undergoes ring-opening polymerization of C=C bonds to effectively inhibit side reactions at the interface [163, 164]. The generated HF promotes the formation of a dense SEI enriched with LiF, enabling uniform Li deposition and high CE [165]. By combining the addition of FEC and LiNO_3_, Zhang’s group successfully achieved 83% capacity retention after 150 cycles in a Li metal battery with 33 μm ultrathin Li anode, high-loading LiNi_0.5_Co_0.2_Mn_0.3_O_2_ cathode (4.4 mAh cm^−2^) and lean electrolytes (6.1 g Ah^−1^). The obtained SEI, featuring abundant heterogeneous grain boundaries composed of LiF, LiN_x_O_y_ and Li_2_O, effectively facilitates the rapid diffusion of Li-ions and ensures uniform deposition of Li.

#### Fully Fluorinated Electrolytes

Increasing the fluorination degree of electrolyte is generally considered to stabilize LMBs due to its high reactive stability and LiF-enriched SEI formation [166, 167]. Wang’s research group reported an all-fluorinated electrolyte, composed by 1 M LiPF_6_ in a mixture of FEC-FEMC-HFE (2:6:2 by weight), with high CE up to 99.2% of LMAs [168]. By employing fully fluorinated electrolyte components, a dense and highly-fluorinated interphase can be easily formed on both the LMA and high-voltage cathode, effectively suppressing side reactions at the electrode–electrolyte interface and preventing transition metal dissolution. Consequently, it supports exceptional stable cycling of NCM811/Li cells with 90% retention after 450 cycles and LiCoPO_4_ (LCP)/Li cells with 93% retention after 1000 cycles. Besides, substituting alkyl groups with fluorine atoms can impede the propagation of oxygen radicals during combustion, providing additional nonflammability advantages. In 2020 and 2021, Xue et al. respectively reported the electrolyte solvents FSA [169] and DMTMSA [170], which contain fluorosulfonyl (–SO_2_F) and trifluoromethyl (–CF_3_) groups. Their studies demonstrated that these groups are primarily responsible for the formation of a LiF-rich SEI layer.

#### High Concentration Electrolytes

In most non-aqueous Li-ion conducting electrolytes, the standard concentration is 1 M due to the balance between the number of charge carriers and the ionic mobility of these charge carriers [171, 172]. Solvent molecules solvate Li-ions, resulting in the formation of solvent-separated ion pairs (SSIPs) structure (Fig. 6e) [173, 174]. The structure of the solvation shell is determined by the nature of the solvent and the salt anion. Furthermore, a significant amount of uncoordinated solvent exists beyond the first solvation shell. When the concentration of lithium salt exceeds 3 M, the interaction between Li-ions and anions/solvent molecules in the electrolyte becomes more intense, leading to a solvation structure dominated by contact ion pairs (CIPs) formed through the interaction of Li-ions and anions, as well as aggregates (AGGs) formed by Li-ions, anions, and solvent molecules (Fig. 6f) [175, 176]. In this state, nearly all solvating-solvent molecules must coordinate with the cation of Li salt. Notably, high concentration electrolytes (HCE) exhibit distinct physical and electrochemical properties compared to conventional diluted electrolytes [171]: (1) High thermal stability, the solvent molecules are all coordinated with Li-ions, so that additional energy is needed to disrupt the solvation structure during volatilization; (2) High Li-ion transference number, anions predominantly bind to Li-ions in CIPs and AGGs forms rather than migrating independently under electric field influence; (3) High chemical stability, reduced availability of free solvent molecules enhances competitive reduction of anions leading to formation of dense inorganic SEI layer [44].

Qian et al. [177] demonstrated the utilization of HCE, consisting of 4 M LiFSI in 1,2-dimethoxyethane (DME), which enables a high CE up to 98.4% without dendrite growth even at a high current density of 4 mA cm^−2^. In conventional dilute electrolytes, the solvent tends to react with the plated metallic Li, leading to reduced utilization efficiency. However, in this study, they successfully obtained nodular and compact Li metal with a metallic luster during the high-rate Li plating/stripping process on Cu electrode surfaces, significantly enhance the reversibility of LMA. The SEI layer generated from highly concentrated 4 M LiFSI-DME electrolytes exhibits high conductivity and compactness, which stabilizes the voltage profiles during cycling and prevents further corrosion of the Li metal electrode. Hence, it has achieved stable cycling for 6000 cycles at 4 mA cm^−2^. Moreover, by employing carbonate-based HCE composed of 10 M LiFSI-EC/DMC as an alternative approach, it is possible to transform loose and dendritic lithium deposition morphology into more compact and spherical structures while significantly improving CE from 86% in dilute solutions to an impressive value of 99.6% [178]. The presence of high concentrations FSI^–^ anions facilitate the formation of interphases rich in fluorine content on both LMA and Ni-rich NCM cathode surfaces under aggressive chemical conditions, enabling successful implementation of NCM622/Li cells. (Fig. 6i). In addition, the unique solvation structure of HCE enables highly reactive organic solvents such as sulfones [179], nitriles [180], and phosphate esters [181] to be utilized in LMBs, thereby significantly expanding the range of available electrolyte solvents.

The utilization of HCEs can significantly enhance the reversibility of LMA during cycling, representing a crucial advancement towards practical applications of LMBs. However, the high concentration of lithium salt in HCEs inevitably leads to increased electrolyte viscosity, reduced ionic conductivity, and inadequate wettability towards porous electrodes (Fig. 6h) [182]. Consequently, this elevates the internal resistance of batteries, thereby adversely affecting their charging/discharging performance under low temperatures or during high-energy power usage [176]. To overcome these limitations associated with HCEs, a straightforward approach is to dilute them with other solvents. This strategy aims to strike a balance between ionic conductivity and electrochemical stability while reducing the consumption of lithium salt and minimizing electrolyte costs (Fig. 6g). Therefore, fluoroethers or fluoroalkanes such as 1,1,2,2-tetrafluoroethyl-2,2,3,3-tetrafluoropropyl ether (TTE) and bis(2,2,2-trifluoroethyl) ether (BTFE) have been discovered and utilized for diluting HSEs [183, 184]. The added cosolvent is miscible with major solvents used in HCEs without interfering with Li salts dissociation or coordination. This results in the formation of localized high concentration electrolytes (LHCEs). Ren et al. employed an ether-based 1LiFSI-1.2DME-3TTE (molar ratio) electrolyte for high-voltage NCM811/Li cells[185]. The LHCE could greatly enhance the stability of Ni-rich NMC811 cathode under 4.5 V and enable highly reversibility of LMA with 99.7% CE for 300 cycles. The assembled NCM811/Li coin cells (4.2 mAh cm^−2^) were able to maintain over 80% capacity retention after 150 stable cycles under highly challenging conditions (50 μm Li and 3 g Ah^−1^ electrolyte). This study showcases unprecedented electrochemical performance achieved through optimized electrolyte design using LHCE strategy and establishes its potential to advance practical high-energy LMBs.

Whether employing novel additives, fluorinated electrolytes, or HCEs, researchers are essentially capitalizing on the heightened reactivity of LMA and deliberately shaping the SEI interphase. However, the resultant SEI layer formed in this manner often exhibits a thin thickness of only tens of nanometers, rendering it incapable of accommodating significant volume changes and necessitating continuous consumption of lithium salt or additives to maintain SEI stability.

### Separator Modification

**Fig. 7:**
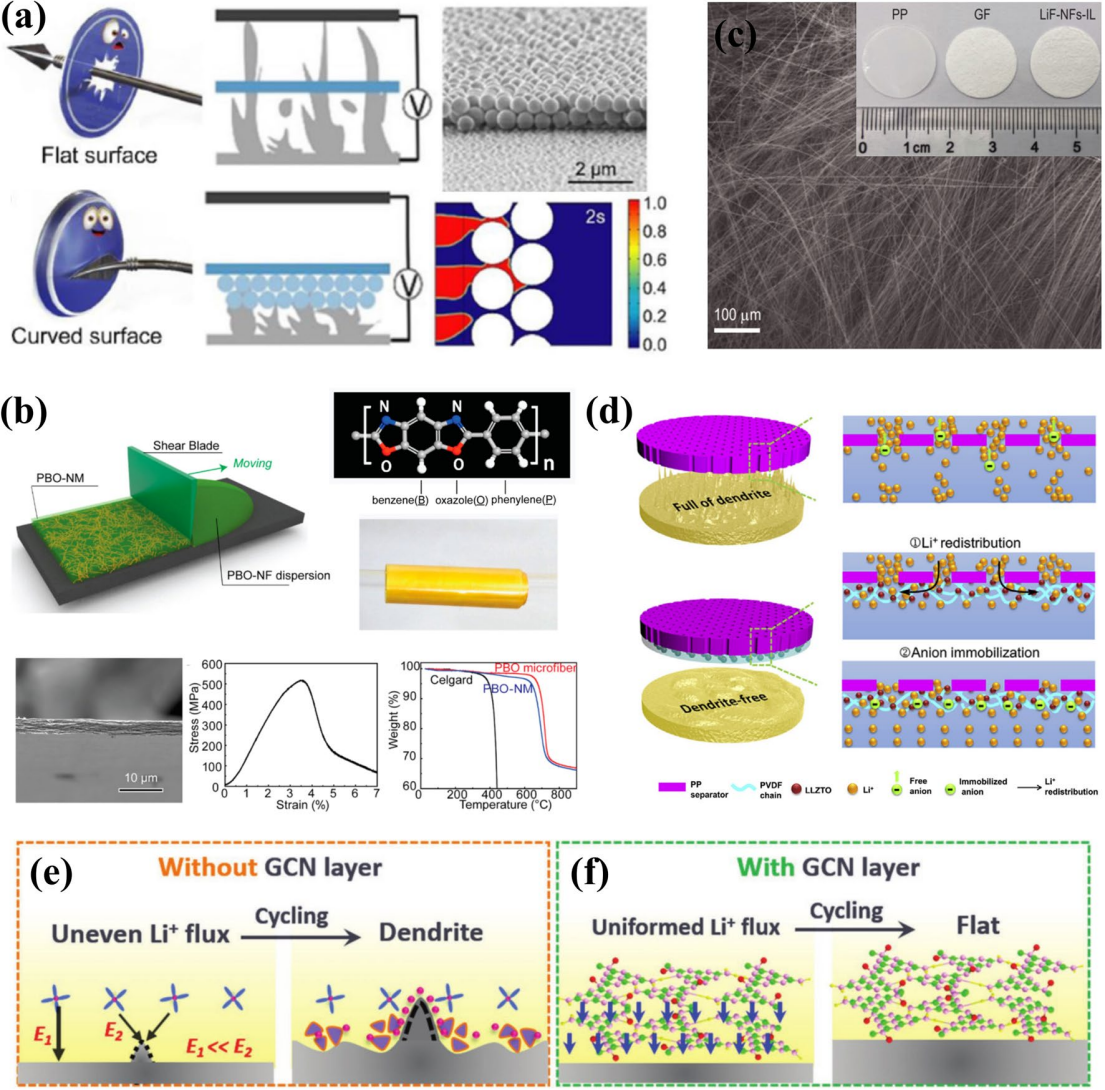
The separator modification for LMAs. **a** Schematic illustration of nano-shield design for separators to resist Li dendrite formation. Reproduced with permission from [188] Copyright 2020, Wiley–VCH. **b** Fabrication process and characterization of a PBO nano-porous separator membrane. Reproduced with permission from [191] Copyright 2016, American Chemical Society. **c** SEM images of LiF-fiber-woven interlayer. Reproduced with permission from [195] Copyright 2022, Oxford University Press. **d** Schematic illustrations of Li deposition behaviors through PP separator and PP-PVDF-LLZTO composite separator. Reproduced with permission from [194] Copyright 2019, Elsevier. Schematic illustrations of Li deposition and corresponding mechanisms **e** without and **f** with GCN layer modification. Reproduced with permission from [198] Copyright 2019, Wiley–VCH.

The separator plays a vital role in lithium batteries by isolating the cathode and anode, ensuring complete electrolyte infiltration, facilitating rapid Li-ion transport, and eliminating electron transfer. Further functional modification of separators can enhance electrochemical performance and safety without adding significant weight or volume. This modification aims to block Li dendrite growth, distribute Li^+^ transport more effectively, and regulate Li deposition [186, 187]. One straightforward way is to coat a rigid layer of inorganic nanoparticles onto the surface to physically impede Li dendrite penetration. Zhu et al. demonstrated that curved surfaces can effectively mitigate the piercing of sharp tips by distributing interfacial stress, and proposed a nano-shield design utilizing SiO_2_ nanospheres coated separator for LMBs (Fig. 7a) [188]. Due to the curved surface effect and the increased growth pathways for Li dendrites resulting from the formation of narrow channels, the nano-shield protected separator can effectively suppress Li dendrite growth. Moreover, it significantly extends the lifespan of charging tests by over 110 h without causing short-circuits at 0.5 mAh cm^–2^ more than five times longer than achieved with blank separators. Similarly, the same effect can be achieved by employing a rigid modification layer consisting of polystyrene (PS) nanospheres, Al_2_O_3_ [189] and AlN [190] nanoparticles. Sun et al. prepared an ultra-thin separator comprising exfoliated nanofibers of poly(*p*-phenylene benzobisoxazole) (PBO), serving as a substitute for commonly used materials such as Polyethylene (PE) and polypropylene (PP) [191]. As shown in Fig. 7b, the nano-porous separator membranes were fabricated via a scalable blade casting process using PBO nanofibers, resulting in a thickness of 3.1 μm, high tensile strength of 525 ± 20 MPa, and thermal stability up to 600 °C. With such high-performance separators, it remained “dendrite-free” morphology over 700 h cycling and exhibits stable operating at 150 °C.

The distribution of Li-ions can be regulated by the functionalized separators. Wu et al. developed a PP separator with polyacrylamide-grafted graphene oxide molecular brushes to improve electrolyte wettability and distribute the Li^+^ flux in molecular level [192]. As a result, LMA demonstrated long-term reversible plating/stripping for over 2,600 h at a 2 mA cm^−2^, along with uniform Li deposition with high CE of 98%. The presence of ceramic particles exhibiting fast Li-ion conductivity not only facilitates the uniform transport of Li-ions, but also mechanically suppress Li dendrite growth due to their high Young’s modulus [193]. Huo et al. fabricated a composite separator by coating PVDF and Li_6.4_La_3_Zr_1.4_Ta_0.6_O_12_ (LLZTO) on a commercial PP separator [194]. The abundant interface between PVDF and LLZTO provides a 3D Li^+^ transport channel, while the composite separator further immobilizes anions, promoting even distribution on the Li anode as shown in Fig. 7d. The synergistic effects result in higher Coulombic efficiency and steady cycling for LMBs. Due to its exceptional innate properties in Li^+^ distribution and deposition, a unique LiF interlayer woven by millimeter-level, single-crystal LiF nanofibers has been designed to enable dendrite-free and highly reversible Li deposition (Fig. 7c) [195]. The presence of LiF nanofibers creates continuous pathways for interfacial Li^+^ transport pathways, renders lower nucleation and high migration energy barriers, resulting in low nucleation and high migration energy barriers that promote uniform plating and stripping processes. This ultimately enables steady cycling in symmetric Li/Li cells for over 1600 h at a plating/stripping depth of 4 mAh cm^−2^.

The deposition behavior of Li is correlated with the selective functional groups that are attached to separators [196, 197]. Guo et al. employed an auto transferable strategy to modify PP separator with graphene-like carbon nitride (GCN) without the need for inert atmosphere protection[198]. The GCN-modified separator provides abundant N-group sites, facilitating the formation of transient Li-N bonds in the electrolyte prior to Li deposition. This subsequently results in a significantly reduced energy barrier for deposition. Typically, Li^+^ tends to accumulate at the tips of deposited Li due to their shorter diffusion pathways and stronger electric field, which exacerbate the growth of Li dendrites (Fig. 7e). In contrast, by introducing a GCN layer, it serves as a pre-stabilization agent for Li^+^ near the LMA surface through transient Li-N bonding interactions, thereby tailoring the distribution of Li^+^ during cycling and promoting a much denser and smoother deposition morphology (Fig. 7f). As a result, Cu/Li cells assembled in this manner achieve over 900 cycles with a CE exceeding 99%, effectively suppressing the growth of Li dendrites. Recently, Liu et al. designed a self-assembled monolayers (SAMs) onto Al_2_O_3_-coated PP separator [Al_2_O_3_–OOC(CH_2_)_2_X], and explored the effect of different terminal functional groups (X = NH_2_, COOH) on Li deposition [199]. It was discovered that the polar carboxyl group (–COOH) linkage could generate strong dipole moments, thereby expediting the degradation kinetics of C-F bond in LiTFSI and promoting the formation of LiF-enriched SEI. Hence, this enhanced surface chemistry facilitated rapid Li^+^ transfer and effectively suppressed dendritic Li growth, leading to extended lifespan in symmetric Li/Li cells with steady cyclability for over 2500 h at a small overpotential of 40 mV under a current density of 1 mA cm^−2^. Notably, even under stringent conditions (N/P ratio = 3, electrolyte volume is 60 μL), LFP/Li full cells demonstrated an enhanced lifespan of more than 450 cycles with a capacity retention above 80% and an average CE above 99.9%. This work highlights the influence of functional groups on Li^+^ distribution and metallic Li deposition behavior, emphasizing the significance of separator modification for advancing LMBs.

The deposition behavior of metallic Li can be readily adjusted by modifying the separator, which involves manipulating the process of Li-ion diffusion and desolvation. This approach represents a crucial enhancement strategy with significant implications for improving battery performance.

### Alloyed Anodes

**Fig. 8:**
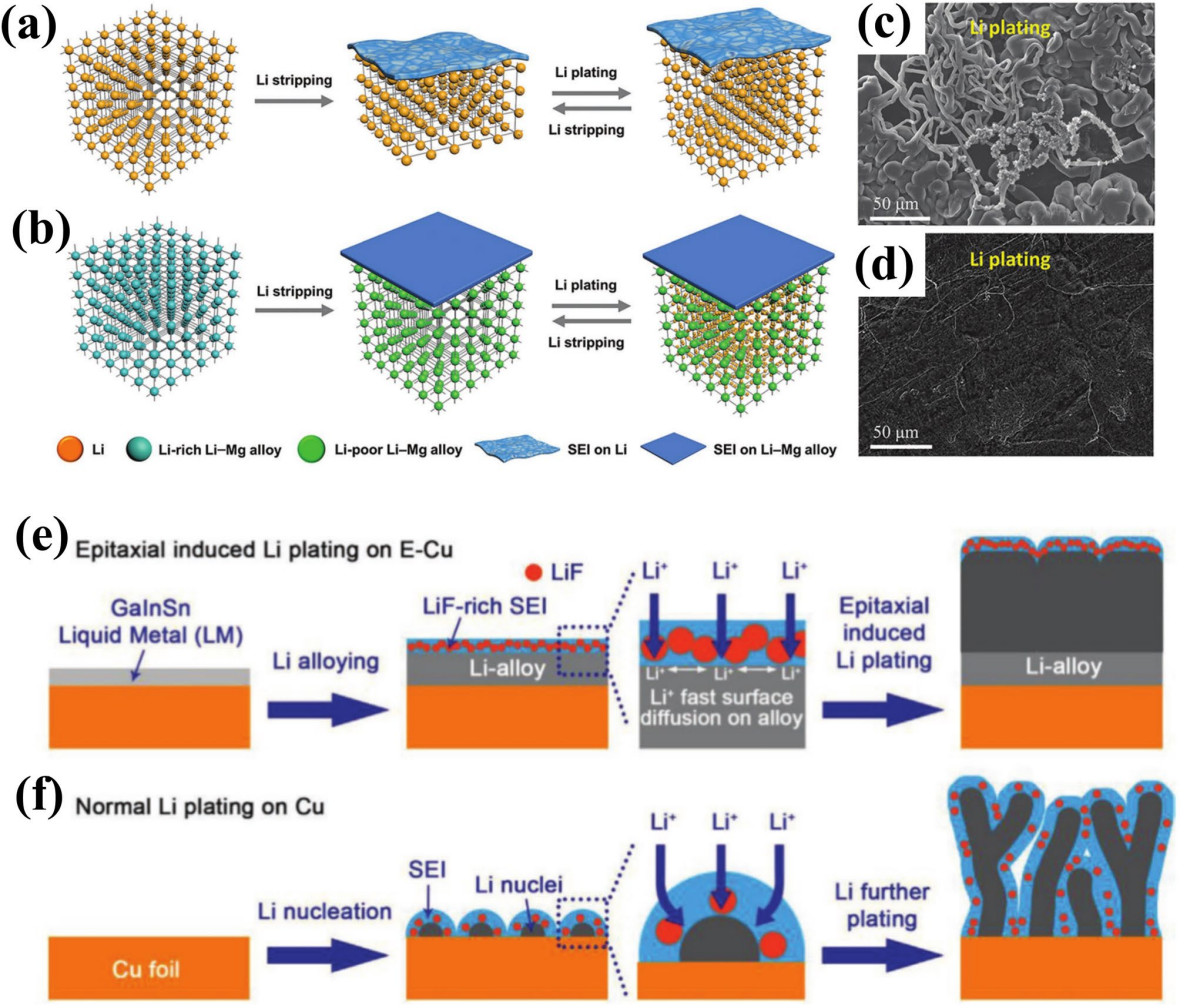
The alloyed anode design for LMAs. Schematic illustration of **a** bare Li and **b** Li-Mg alloy anodes during Li stripping/plating process. SEM images of **c** bare Li and **d** Li-Mg alloy anodes after Li plating at 0.5 mA cm^−2^ for 24 h. Reproduced with permission from [207] Copyright 2019, Wiley–VCH. Schematic illustration of Li deposition behavior on **e** Cu current collector and **f** Ga-In-Sn liquid metal (LM). Reproduced with permission from [209] Copyright 2021, Wiley–VCH.

The use of Li-rich alloy as an anode can significantly reduce the nucleation overpotential and guide uniform Li deposition, while maintaining a stable 3D skeleton structure to mitigate volume expansion during cycling [200]. Therefore, Li-B [201], Li-Mg [202], Li-In [203], and Li-Al [204, 205] have been successively utilized to enhance the electrochemical performance of LMBs [206]. Kong et al. investigated Li-Mg alloy with high Li content of 81.43 wt% as an anode for Li–S batteries, to stabilize the bulk and the surface of LMA (Fig. 8a, b) [207]. This selective alloyed anode can form a nano-porous sponge structure after delithiation, ensuring the structural integrity of the anode, which is beneficial for reducing side reactions and maintaining smooth surface morphology during cycling (Fig. 8c, d). Meanwhile, it provides a mixed electron/ion conductive interface that facilitates Li-ion diffusion and electroplating. Furthermore, Gao et al. studied the effect of Mg amount in Li-Mg anode by density functional theory (DFT) calculations [208]. The Li-Mg alloy with approximately 5 wt% Mg was found to exhibit the lowest Li absorption energy when each Mg atom dominates all surface areas, resulting in a smooth and continuous deposition of Li on the surrounding surface centered around the Mg atom. Combining the use of LHCE, resulting alloyed anode exhibited superior cycling stability in high-loading cathode (4.2 mAh cm^–2^) and lean-electrolyte (3 g Ah^−1^) LMBs operating at 4.4 V. Han et al. synthesized Li alloy anodes via Li thermal reduction of metal ethoxides (Al (EtO)_3_) to obtain nanoscale Li-Al alloys with uniform distribution, where the mass of Al accounts for 20% [205]. Compared to bare Li anodes, the in-situ formed Li-Al anodes can reduce the nucleation potential and provide additional fast ion diffusion channels, leading to ultralow overpotential (5 mV) and ultralong lifetime (1100 h) under the current density of 0.5 mA cm^−2^. When coupled with NCM622 cathode (21.6 mg cm^−2^), it demonstrated stable cycling for 300 h and steady CE of 99.5%.

It is worth noting that the epitaxial growth of metallic Li can be achieved by utilizing a Ga-In-Sn (mass ratio of 68.5:21.5:10) liquid metal (LM) coating layer on Cu electrode, effectively suppressing the formation of Li dendrites (Fig. 8e, f) [209]. On one hand, the functional LM layer initiates Li storage through alloying reactions, forming an epitaxial induced layer and regulating the growth direction of Li dendrites. On the other hand, it enhances the surface diffusion ability of Li-ions and effectively prevents excessive Li deposition at a fixed site to smoothen out the morphology of LMA surface. Considering the preferential decomposition of LiFSI in ether-based electrolyte to stimulate LiF-rich SEI, an assembled anode-free pouch cell can deliver high-capacity retention of 84% after 50 cycles under harsh conditions (with a cathode mass loading of 25 mg cm^−2^ and electrolyte addition of 2 g Ah^−1^) to realize remarkable energy density of 420 Wh kg^−1^. Moreover, leveraging the intrinsic healing ability of LM, Zhou et al. proposed a sustainable and repairable lithium alloy anode [210]. This alloyed anode can also enable the reconstruction of broken interface and the elimination of Li dendrites through solid-to-liquid healing conversion, significantly enhancing anode survivability. As a result, it has demonstrated an ultralong cycle life exceeding 1300 cycles at a capacitance level as high as 5 mAh cm^−2^ through two healing processes, while achieving an unprecedentedly high discharge current density of up to 25 mA cm^−2^ with a capacity as high as 50 mA h cm^−2^.

### External Field Regulation

**Fig. 9:**
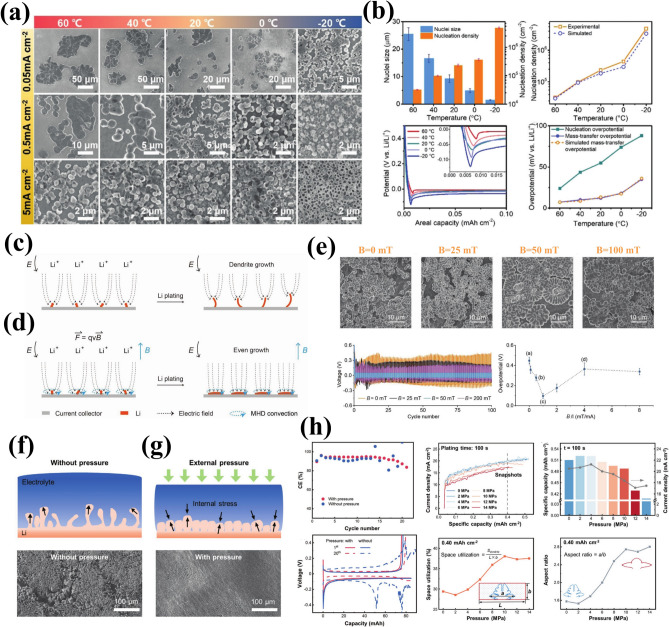
The external field regulation for LMAs. **a** SEM images of Li nuclei under different temperature conditions. **b** Comparison of Li nuclei size, nucleation density, nucleation overpotentials and mass-transfer overpotentials under different temperature conditions. Reproduced with permission from [51] Copyright 2019, Wiley–VCH. Schematic illustration **c** without and **d** with external magnetic field to show the elimination effect of tip Li dendrite growth by Lorentz force. Reproduced with permission from [213] Copyright 2019, Wiley–VCH. **e** The variation of surface morphology and electrochemical performance under different magnetic fields. Reproduced with permission from [214] Copyright 2019, Wiley–VCH. Schematic illustration of the morphology of Li dendrites **f** without and **g** with external pressure to show the impact of external pressure on LMAs. h The analysis results of LMAs under 0–14 MPa external pressure. Reproduced with permission from [216] Copyright 2021, Wiley–VCH

Apart from the inherent properties and electrochemical reactions, external field conditions exert varying degrees of influence on the entire process of Li deposition, encompassing Li^+^ diffusion in electrolyte, interfacial Li^+^ reduction and nucleation, Li bulk phase diffusion and interface migration [211]. Among these influencing factors, temperature is an inevitable concern. Research conducted by Cui’s group has demonstrated that elevating the temperature to 60 °C significantly enhances the reversibility of LMA with an average CE of 99.3% over more than 300 stable cycles. In contrast, at 20 °C, there is a drastic drop in CE within only 75 cycles accompanied by an average CE of only 90.2% [212]. Cryo-electron microscopy reveals a significantly distinct SEI structure at 60 °C, which exhibits good mechanical stability and effectively suppresses the continuous occurrence of parasitic reactions, ensuring superior cycling stability. Yan et al. [51] furtherly investigated the relationship between nucleation overpotential, nuclei size, nucleation density and diffusion coefficient with temperature through ex situ and in situ microscopy characterizations (Fig. 9a). As the temperature increased from − 20 to 60 °C, the Li nucleation overpotential decreases, leading to an increase in nuclei size and a decrease in nucleation density. This is beneficial for compact Li deposition and avoiding excessive exposure to electrolyte. Besides, the elevated temperature promotes the diffusion of Li^+^ aprotic electrolyte due to the enhanced lithiophilicity and the increased Li-ion diffusion coefficient, thereby contributing to dendrite-free Li growth behavior at high temperatures (Fig. 9b).

However, the intense heat generated during cycling poses challenges in controlling the internal temperature, and prolonged operation at high temperatures can limit the calendar life of lithium batteries. Luo et al. introduced a magnetic field into Li plating process and established the relationship between current density and magnetic flux intensity[213]. When an electric field (E) was coupled with magnetic field (B), the movement of charged particles is influenced by Lorentz force ($F=qvB$), where $F$ is Lorentz force, $q$ denotes the quantity of electric charge for moving particles, $v$ signifies the velocity of charged particles, and B indicates magnetic flux intensity exerted on the lithium battery. The Li-ions experience spiral motion known as magnetohydrodynamics (MHD) effect under Lorentz force from electromagnetic fields [214]. This stirring process of MHD effect disperses concentrated Li-ions at protuberances caused by uneven electric field distribution, thereby reducing diffusion layer thickness and enhancing mass transport near the anode surface (Fig. 9c, d). Consequently, the deposition sites for Li^+^ are expanded and electrodeposition on the current collector is uniformly distributed, successfully mitigating uncontrollable growth of Li dendrites and uneven topography formation on LMA surface. Furthermore, the electrochemical performance exhibits a strong correlation with the intensity of the magnetic field, specifically demonstrating a funnel-shaped relationship between overpotential and B/I ratio at different current densities (Fig. 9e). Based on these findings, Wang et al. proposed a magnetic Fe_2_CoAl alloy on carbon paper (Fe_2_CoAl/C) [215]. The Fe_2_CoAl/C host not only exhibits favorable lithiophilic characteristics with reduced overpotentials but also promotes the uniform distribution of Li^+^. Under such synergistic effects, the assembled symmetrical Li/Li cells show stable cycling up to 1000 h at 1 mA cm^−2^ in ester-based electrolyte, and the LFP full cells show high coulombic efficiency of 99.36% after 900 cycles at 1 C.

The role of external pressure in the Li deposition process is unconventional. According the mechano-electrochemical phase field model proposed by Zhang’s group, two main influences are outlined [216]: (1) Inhibiting the progress of Li electroplating reaction; (2) Physically shaping deposited Li to dense and smooth morphology (Fig. 9f, g). Essentially, there exists an optimal pressure that can overcome the side effects of hindering electroplating and causing mechanical instability (Fig. 9h). Jiang and colleagues confirmed the presence of stress concentration during Li dendrite growth and developed a soft polydimethylsiloxane (PDMS) substrate for wrinkling-induced stress relaxation, which mitigates the driving force behind Li dendrites [217]. By employing a stress-release substrate, high CE more than 98% for over 200 cycles of LMA and superior cyclic stability with greater than 99.5% coulombic efficiency in LFP/Li cells can be achieved. With the use of a stress-releasing substrate, it has achieved high CE exceeding 98% for over 200 cycles of LMA, as well as superior cyclic stability with greater than 99.5% CE in LFP/Li cells.

Additionally, the impact of other external factors, such as pulse current [218, 219], acoustic wave [220] and supergravity [221] have been thoroughly investigated and effectively utilized to regulate the deposition behaviors of metallic Li. These external field factors continue to exert their influence by altering the kinetics of the electrochemical processes. Therefore, an in-depth exploration will contribute to comprehending the formation mechanism of lithium dendrites and pave the way for dendrite-free LMAs.

## Transition from Liquid to Solid Electrolyte

**Fig. 10:**
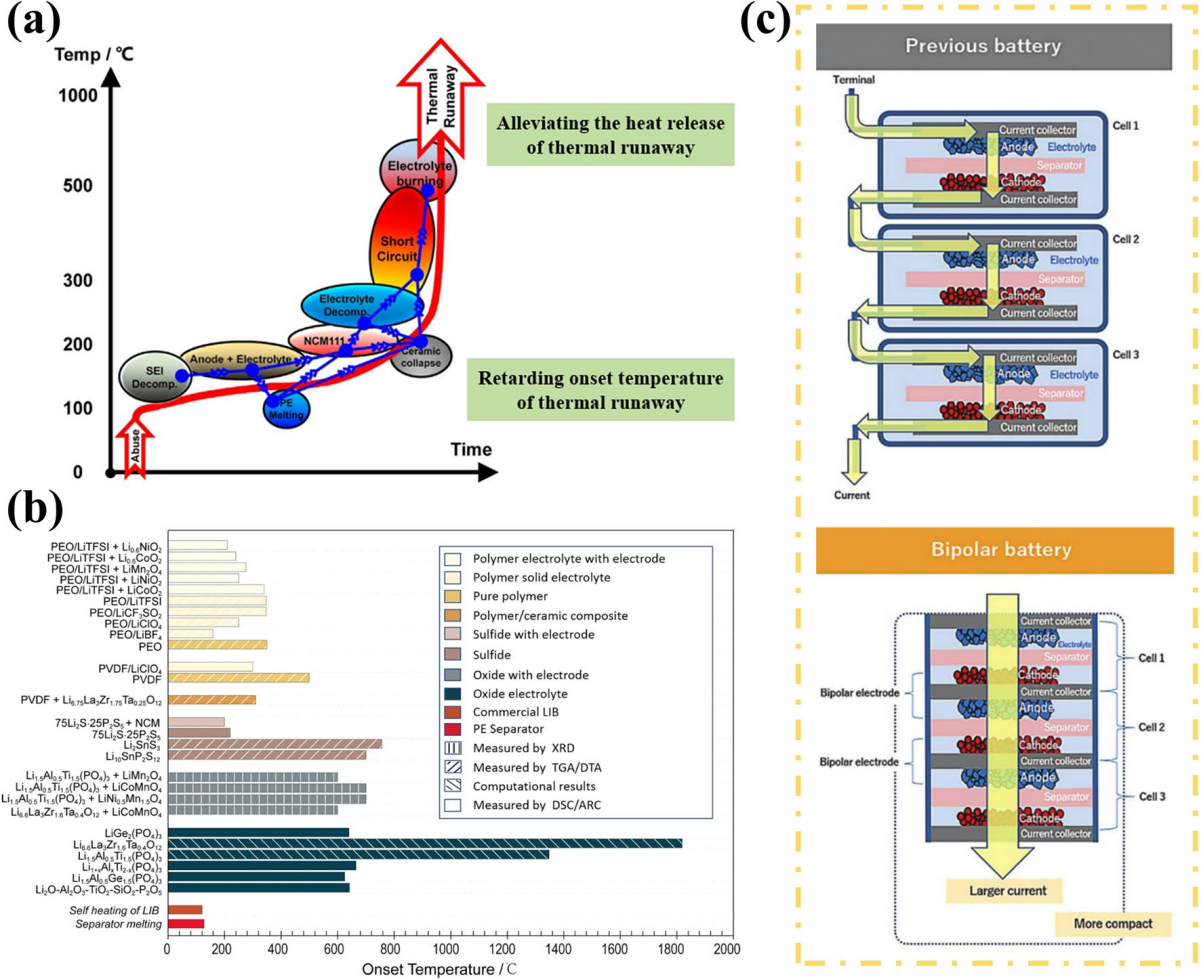
Advantages of the transition from liquid to solid electrolyte in LMBs. **a** Merits of solid-state electrolyte (SSE) over organic liquid electrolyte for battery thermal safety. Reproduced with permission from [222] Copyright 2017, Elsevier. **b** Comparison of onset temperature of exothermal behaviors for different SSEs. Reproduced with permission from [224] Copyright 2020, American Chemical Society. **c** Schematic illustration of packed structure reformation in bipolar-stacked battery for solid-state lithium metal batteries (ASSLMBs). Reproduced with permission [17]. Copyright 2022, Royal Society of Chemistry

From a thermodynamic perspective, the highly reactive LMA inevitably leads to increased heat generation due to intensified potential chemical reactions with liquid electrolytes and cathode materials. This poses a significant threat to the safety and reliability of lithium batteries, particularly for electric vehicles. Thermal runaway (TR) is a critical scenario characterized by an abrupt and intense release of heat, often accompanied by violent combustion, forceful ejection of materials, or even explosion [222]. It represents a major safety concern for lithium batteries as it involves a series of exothermic reactions spanning low to high temperatures. These reactions are followed by SEI decomposition, collapse of PP/PE separator resulting in internal short circuiting, oxygen release from the cathode, intense reaction between the cathode and anode, and combustion of liquid electrolytes etc. [223]. It is generally believed that SSEs have inherent characteristics capable to delaying the onset temperature of TR and moderating the propagation of heat propagation [222], as shown in Fig. 10a. Firstly, SSEs can significantly lower the reaction kinetics with cathode and anode, remarkably reduce both the amount and rate of heat generation at the early stage of TR process. Secondly, SSEs exhibit superior thermal stability and mechanical strength, effectively breaking the reaction chain and reducing heat release during intensified exothermic side reactions at elevated temperatures. In contrast, separators based on PP or PE-based separator are prone to be melting below 155 °C, resulting in direct contact between the cathode and anode and triggering significant heat release. Thirdly, due to their inherent non-flammability, non-volatility and immobility characteristics, SSEs can mitigate violent burning and jet combustion events associated with TR risks. Figure 10b presents a summary of the thermal behaviors of various SSEs at elevated temperature [224]. Among them, oxide-based electrolytes exhibit the best thermal stability owing to their high initial oxygen loss and thermal deposition temperature, which can reach up to 1360 and 1800 °C for Li_1.5_Al_0.5_Ti_1.5_(PO_4_)_3_ (LATP) and Li_6.6_La_3_Zr_1.6_Ta_0.4_O_12_ (LLZTO), respectively. Except for certain sulfide electrolytes that may undergo crystallization at temperatures exceeding 200 °C, most sulfide SSEs are relatively stable [225, 226]. For instance, the exothermic reaction onset temperature of Li_10_SnP_2_S_12_ is up to 700 °C [227]. Although incorporating Li salts reduces the decomposition temperature in polymer electrolytes, their thermal stability still surpasses that of liquid electrolytes [228].

In liquid electrolytes, the diffusion of soluble electrode components can result in transition metal leaching in LIB cells and “polysulfide shuttle effect” in lithium-sulfur cells, commonly known as “cross-talk” [229]. The use of SSEs can eliminate this unwanted phenomenon and enable independent reactions for cathode and anode [230]. Additionally, transitioning from a liquid to a solid-state electrolyte facilitates the formation of a compact structure through bipolar stacking [17]. Consequently, the cathode of one cell is laminated onto the backside of the current collector of the subsequent cell, allowing for multiple cells to be densely packed into a smaller area with reduced packaging and circuit resistance (Fig. 10c). This leads to a significant enhancement in both energy and power density within the pack [231]. Bipolar stacking is typically unfeasible with liquid electrolytes due to the potential for electrolyte leakage and resulting ionic short circuits.

In conclusion, considering the inherent advantages of SSEs in terms of enhanced safety, improved thermal and chemical stability, as well as simplified packaging structure, the transition from liquid to SSEs represents an inevitable progression for LMBs.

## Fundamental Issues for LMB in Solid Electrolytes

**Fig. 11:**
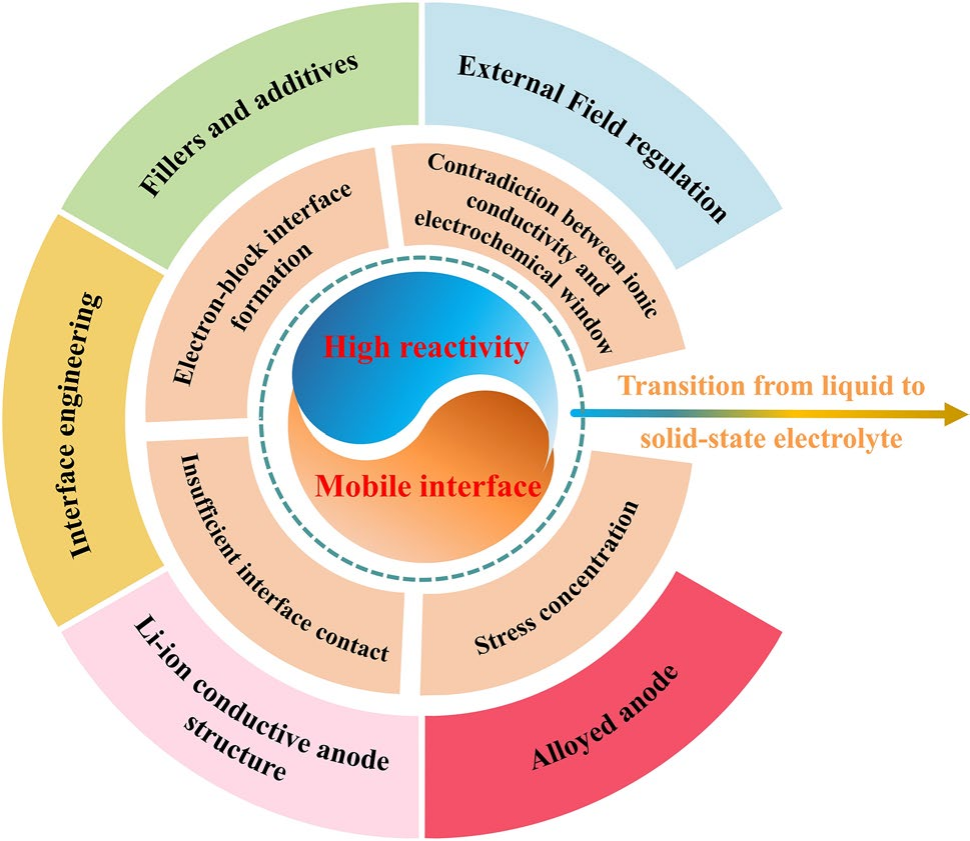
Schematic illustration of evolution of fundamental issues and corresponding strategies in lithium metal batteries (LMBs) from liquid to solid-state electrolyte

Compared to liquid electrolytes, SSEs can evidently impair the interfacial chemical reaction activity. Moreover, their inherent rigidity and the creation of an inhomogeneous interface present additional challenges. These fundamental issues are summarized in Fig. 11, encompassing the contradiction between ionic conductivity and electrochemical window, formation of electron-block interface, insufficient interface contact, and stress concentration.

### Contradiction Between Ionic Conductivity and Electrochemical Window

**Fig. 12:**
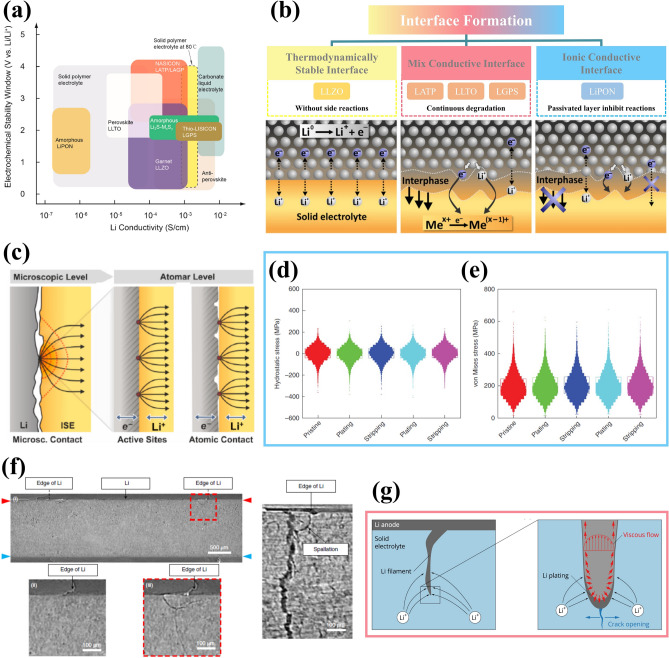
Fundamental issues for LMA in ASSLMBs. **a** Electrochemical window and ionic conductivity for different SSEs. Reproduced with permission from [224] Copyright 2020, American Chemical Society. **b** Schematic illustration of three types of interfaces between SSEs and LMA. Reproduced with permission from [267] Copyright 2015, Elsevier. **c** Schematic illustration of current constriction phenomenon due to macroscopic insufficient contact, low active site concentration and loss of atomic contact during cycling. Reproduced with permission from [100] Copyright 2019, Wiley–VCH. The distribution of stress response in polycrystalline LLZO electrolyte for **d** hydrostatic stress and **e** von Mises stress. Reproduced with permission from [289] Copyright 2022, The Authors, published by Springer Nature. **f** The initialization and propagation of cracks and the propagation of Li dendrites. Reproduced with permission from [291] Copyright 2021, The Authors, published by Springer Nature. **g** Schematic illustration of stress distribution and Li filament growth. Reproduced with permission from [294] Copyright 2019, IOP Science

Over the past few decades, a series of diverse SSEs has been discovered. Based on their chemical composition and molecular structure, they can be broadly classified into oxides, sulfides, polymers, halides, and thin-film type. As shown in Fig. 12a, sulfide electrolytes are typically characterized by the highest ionic conductivity. For instance, Kamaya et al. reported an extremely high room temperature (RT) conductivity of 12 mS cm^−1^ for a lithium superionic conductor Li_10_GeP_2_S_12_ (LGPS), which is comparable to that of most liquid electrolytes [232]. Substituting the expensive Ge with Sn element maintains the synthesized Li_10_SnP_2_S_12_’s ionic conductivity at 7 mS cm^−1^ while reducing costs to only one third that of LGPS [227]. By utilizing various elements doping, the ionic conductivity of the fabricated Li_9.54_Si_1.74_P_1.44_S_11.7_Cl_0.3_ is furtherly enhanced to 25 mS cm^−1^, which currently stands as the highest among SSEs [233]. This is because, in comparison with O^2−^ ions, S^2−^ ions possess a larger ionic radius and smaller electronegativity, resulting in weaker binding ability to Li^+^ and offering larger ion transport channels [16, 234]. As a result, sulfide electrolytes exhibit significantly superior ionic conductivity especially compared to oxide electrolytes. Nevertheless, the weak binding of Li also leads to inferior antioxidant and anti-reduction performance. LGPS has a narrow electrochemical window ranging from 2.1 to 2.4 V versus Li/Li^+^ [68]. The Argyrodite-type Li_6_PS_5_X (X = Cl, Br, I) electrolyte has recently garnered considerable attention due to its facile synthesis, adequate ionic conductivity, and improved electrochemical stability [235–237]. Upon annealing treatment, the synthesized Li_6_PS_5_Cl (LPSC) and Li_6_PS_5_Br exhibit RT conductivities of 1.9 × 10^−3^ and 6.8 × 10^−3^ S cm^−1^ respectively. However, LPSC still undergoes reduction decomposition to form P, Li_2_S, LiCl, etc., when the potential is lower than 1.71 V [238]. Such a limited electrochemical window restricts the compatibility of sulfide electrolytes with LMA and high-voltage cathodes [239].

While in oxide solid electrolytes, the opposite holds true. Following hetero-valent doping, perovskite Li_3x_La_2/3-x_TiO_3_ (LLTO), garnet Li_7_La_3_Zr_2_O_12_ (LLZO), Na superionic conductor (NASICON) type Li_1+x_Al_x_Ti_2-x_(PO_4_)_3_ (LATP) and Li_1+x_Al_x_Ge_2-x_(PO_4_)_3_ (LAGP) all exhibit an ionic conductivity of approximately 10^−3^ S cm^−1^ at RT, which merely meets the minimum ion transport requirement for lithium batteries [240–244]. In contrast, oxide electrolytes typically exhibit favorable electrochemical stability. For example, LATP and LAGP possess exceptional anti-oxidation properties that make them suitable as coating layers for high-voltage cathodes [245]. The electrochemical window of LLZO spanning from 0.05 to 2.9 V endows it with remarkable stability against metallic Li, only accompanied by very slight interface decomposition [246, 247]. Recently, Tang’s group synthesized a new NASICON-type electrolyte called Li_3_Zr_2_Si_2_PO_12_ (LZSP) using a skeleton-retained cationic exchange approach [248]. LZSP electrolyte exhibits a low activation energy of 0.21 eV and a high ionic conductivity of 3.59 mS cm^−1^ at room temperature, along with a wide simulated electrochemical window ranging from 0.48 to 4.53 V. Whereas, no inorganic SSEs have been identified so far that can simultaneously match the strong reductive LMA and the highly oxidable cathodes.

Following the incorporation of Li salts such as LiTFSI and LiFSI, polymer electrolytes including PEO, poly(vinylidene difluoride-co-hexafluoropropylene) (PVDF-HFP), poly(propylene carbonate) (PPC) and PAN can acquire a certain level of ionic conductivity and facilitate battery operation at 40 ~ 80 °C [249, 250]. Polymer electrolytes generally exhibit superior electrochemical stability, particularly PVDF-HFP and PPC, which can even be coupled with 5 V Spinel LiNi_0.5_Mn_1.5_O_4_ cathodes [251]. They also demonstrate remarkable stability against LMA despite the slight reduction tendency of PEO to generate C_2_H_4_, H_2_ and Li_2_O as reported in some literature [252]. However, the limited ionic conductivity (~ 10^−4^ S cm^−1^ at RT) of dry polymer electrolytes hinders their further application. It is widely recognized that amorphous polymers rather than the crystalline phase predominantly facilitate Li-ion transport [16, 253]. Therefore, it becomes necessary to introduce branching chains or blocks into the polymer matrix or incorporate inorganic nanoparticles to reduce its degree of crystallization for enhancing overall ionic conductivity [254].

Thin-film type Li_2.88_PO_3.73_N_0.14_ (LiPON) electrolyte was initially fabricated by Bates et al. via magnetron sputtering of Li_3_PO_4_ in N_2_ atmosphere and successfully implemented in thin-film batteries with a thickness of several hundred nanometers [255–257]. However, the ionic conductivity of LiPON is only ~ 3 × 10^−6^ S cm^−1^, rendering it unsuitable for direct use in large-sized and high-capacity batteries. By the virtue of excellent compatibility with metallic Li, it is more used as a buffer layer between LMA and other SSEs [258, 259]. Li_a_MX_b_ (X = Cl, Br, I) represents the general structure of halide electrolytes characterized by weak ionic bonding. Li-ions are highly mobile within this anionic skeleton structure, resulting in an ionic conductivity higher than 10^−3^ S cm^−1^ at RT [260–262]. Sun’s team has successively reported high-crystallinity Li_3_ScCl_6_ and Li_3_InCl_6_ halide electrolytes, which exhibit ionic conductivity of 3.02 × 10^−3^ and 1.49 × 10^−3^ S cm^−1^, respectively [263, 264]. These SSEs also demonstrate satisfactory performance when paired with LCO and LiNi_0.83_Mn_0.06_Co_0.11_O_2_ (NMC83) cathodes. Moreover, direct contact between halide electrolytes and LMA can induce severe decomposition reactions, resulting in increased interface resistance and overpotential [265]. This can damage the safety and reversible capacity of lithium batteries. Generally, alloyed anodes such as Li-In and Li-Sn are used for halide or sulfide-based ASSLMs [266].

Due to the conflicting electrochemical performance of various SSEs in terms of their electrochemical window and ionic conductivity, it is challenging for a single SSE to meet all requirements of LMBs. Therefore, besides developing novel SSEs, practical applications of SSEs still rely on the multi-utilization of different types of SSEs, double-layer or multi-layer structures, and interface modifications via buffer layers.

### Electron-block Interface Formation

Due to the unsatisfactory chemical stability of the SSEs and the driving force of the applied potential to electrochemical reactions, three types of interfaces between SSEs and metallic Li may potentially form based on their electronic and ionic properties (Fig. 12b) [267]: (I) thermodynamically stable interface, where SSEs do not react with LMA; (II) mixed conductive interface (MCI), which can simultaneously transport Li-ions and electrons but leads to continuous degradation of the interface; (III) ionic conductive but electronic insulating interface, which can passivate the interface and effectively prevent further parasitic reactions. Type I interface allows decent Li-ion transport while reducing the active Li loss during cycles, often considered as the most ideal interface. It should be noted that LLZO participates in the formation of such an interface. However, establishing a stable interface does not necessarily solve all problems; challenges still exist regarding insufficient interfacial contact and stress concentration.

For type II interface, Li–Ge or Li-Ti alloys are typically formed at the interface with LATP, LAGP, LLTO and LGPS electrolytes, providing additional electronic conductivity. Hartmann and co-workers observed that Li-Ti/Li–Ge alloy contained interlayer with thickness of 20 μm was formed upon contact of LATP and LAGP with metallic Li, where the electronic conductivity was about three orders of magnitude higher than that in the bulk phase [268]. Such MCI undergoes continuous degradation and thickens during cycling, leading to increased impedance and significant polarization. When in contact with LMA, LiPON forms a dense passivation layer consisting of Li_2_O, Li_3_P, Li_3_PO_4_ and Li_3_N to establish a type III interface [269]. This enables a stable cycling for more than 10,000 cycles with coulomb efficiency above 99.98% in assembled LiNi_0.5_Mn_1.5_O_4_ cells [270]. In the absence of avoiding side reactions, it is highly desirable to have a stable passivated layer possessing both high ionic conductivity and electron-blocking properties. By monitoring the dynamic evolution of Li concentration profiles through real-time operando neutron depth profiling, Wang’s group discovered that Li dendrites could directly nucleate and grow inside LLZO and amorphous Li_3_PS_4_ electrolyte [271]. Comparing the electrochemistry of LiPON under different temperatures, it is concluded that high electronic conductivity is responsible for the dendrite formation in these SSEs, highlighting the critical role of type III interface. Through a multiscale modeling approach that integrates DFT calculations and phase field simulations, Qi et al. have discovered that the band gap of surfaces on SSEs is lower than that in bulk phase [272]. This reduced band gap pushes the unoccupied state below the Li-plating potential and facilitates electron transfer from LMA to the surface state. Additionally, by combining neutron microscopic observation of LLZO electrolyte, Liu et al. discovered that half of the grain boundaries exhibit a bandgap 1–3 eV lower than that in bulk LLZO [23]. This creates potential channels for leakage current and can result in premature reduction of Li^+^ by electrons at the grain boundaries, leading to local filament formation and eventual short circuit. Using operando Kelvin probe force microscopy, Berger and co-workers confirmed the consensus that interfacial electronic properties dictate dendrite growth in SSEs [273]. The grain surfaces and grain boundaries of cubic-LLZO are prone to trap excess electrons, which promotes rapid Li growth of Li dendrites along grain boundaries.

### Insufficient Interface Contact

The replacement of rigid SSEs with high modulus will inevitably lead to poor interface contact and reduced active sites for Li deposition, resulting in distortion of electric current lines in the vicinity of LMA. This phenomenon, known as current constriction, occurs when the current is forced to pass through discrete contact spots [274]. In early 1983, Meyer et al. firstly proposed the concept of current constriction as the underlying cause of electrode polarization at the Li_3_N/Li interface [275]. More recently, its impact on lithium dendrite propagation along inorganic SSEs has been further emphasized [276–278]. For LLZO/Li interface, it has been demonstrated that current constriction predominantly contributes to overall polarization in symmetric Li/Li cells [279]. The reasons for insufficient interface contact can be classified as follows (Fig. 12c): (a) macroscopic insufficient contact caused by voids or surface contamination layers; (b) low concentration of active sites resulting from heterogeneities of SSEs; (c) dissolution of Li atoms during the process of Li plating/stripping, which leads to loss of atomic-level contact. The volume change and voids formation have exacerbated the interface contact issues during cycling. By employing operando synchrotron X-ray computed microtomography, Lewis et al. successfully visualized the formation of voids in LLZO and Li_10_SnP_2_S_12_ (LSPS) electrolytes during lithium stripping in symmetric cells, revealing the intricate interplay between interface contact and cell behavior[280]. It is the loss of contact that drives current constriction at the interface between SSEs and LMA, resulting in large interfacial impedance and local electric field. This ultimately exacerbates heterogeneities and promotes dendrites growth at weak points. In addition, Zhang and colleagues have provided a comprehensive understanding of void accumulation evolution during stable, transitional, and failure stages while also uncovering mechanisms behind current density- and areal capacity-dependent void nucleation and growth, offering valuable insights for designing solid–solid interfaces [281].

In short, there is a significant demand for achieving optimal physical contact at the SSE/Li interface, which can be potentially addressed through the removal of surface contaminants, enhancement of molten Li wettability, incorporation of flexible polymer interlayers as buffers, and application of external stress.

### Stress Concentration

According to Newman and Monroe’s prediction, the growth of Li dendrites can be effectively suppressed by SSEs with a sufficiently large shear modulus, which is estimated to be around 9 GPa[282]. Except for soft polymer electrolytes with a Young’s modulus lower than 5 GPa, stiff inorganic solid-state electrolytes are expected to impede the propagation of Li dendrites due to their superior mechanical strength in initial attempts of ASSLMBs [253, 283, 284]. Sulfide electrolytes exhibit a shear modulus that approaches the threshold value, with LGPS and LSPS having shear modulus of approximately 7.9 and 11.2 GPa, respectively [285]. Besides, oxide electrolytes have been reported with an exceptionally high shear modulus nearly eight times larger than the threshold value [286]. However, experimental tests have shown that rigid SSEs cannot eliminate Li dendrite propagation as cells still experience constant short circuits even at much lower current densities below 1 mA cm^−2^ [287, 288]. This observed outcome is attributed to the highly heterogeneous stress state of SSEs at both the interface and grain boundaries.

Dixit et al. combined Far-field high-energy diffraction microscopy (F-HEDM) with X-ray tomography measurements to track the stress evolution of LLZO at grain level during electrochemical cycling [289]. The average hydrostatic and von Mises stress values exhibit negligible variation during cycling across more than 30,000 grains, while these stress values for individual grains are distributed over a wide range of magnitudes (Fig. 12d, e). Only a minority of grains exhibit changes in mean stress, indicating the presence of highly localized heterogeneities within the polycrystalline solid electrolyte. Notably, stresses tend to concentrate at heterogeneous areas such as grain boundaries, defects, and secondary phases. Operando microprobe scanning electron microscopy observations have revealed that Li dendrite intrusion into LLZO primarily occurs at nanoscale cracks [290]. And such microstructure defects can be readily induced by a compressive strain as low as ~ 0.070% either pre-existing or generated via external loading. Under concentrated stress, the crack front propagation is well ahead that of metallic Li [291]. Ning et al. conducted a comparative analysis on the propagation of crack front and Li dendrite growth within a Li/LPSC/Li cell. During the process of Li plating, conical “pothole”-like cracks are generated in the inorganic SSE adjacent to the plated electrode and spread along a path where exhibits higher porosity within the ceramic (Fig. 12f). These cracks tend to occur more frequently at the edges of LMA due to localized electric field and current density effects. Additionally, transverse cracks emerge from these initial cracks. As metallic Li accumulates, it drives further widening and propagation of both initial and transverse cracks. In essence, lithium dendrites follow the path created by these cracks and eventually penetrate through the inorganic SSE, leading to short circuits.

Several models have been proposed to estimate crack propagation and evaluate the endurance of different SSEs under Li dendrite growth [292–295]. Essentially, Griffith’s fracture theory has been utilized to describe crack extension driven by electroplated Li and consequent Poiseuille pressure when a critical internal stress within the flaw is surpassed (Fig. 12g) [288]. In this model, the fracture toughness K_1c_ of inorganic SSEs determines the threshold at which electrochemically induced stress causes fracture. It seems that fiber reinforcement and phase transformation toughening are among the few viable methods for blocking crack propagation and inhibiting the growth of Li dendrites. However, predictions for oxide and sulfide electrolytes do not coincide with experimental results due to high sensitivity of crack propagation to surface flaw geometry [296]. Moreover, cracks significantly impact the interface kinetics and ionic transport within bulk inorganic SSEs [297]. Therefore, a more suitable approach for fracture analysis is an electrochemical-mechanical coupling mechanism based on Butler-Volmer reaction kinetics extended with mechanical effects and incorporating the plastic properties of metallic lithium [295]. On one hand, the mechanical pressure generated by metallic Li filling the cracks can mitigate cathodic current smoothing and deflect electrodeposition away from the crack tip [298]. On the other hand, gradually inserted Li filaments may accumulate to high levels of pressure sufficient to propagate cracks, continuously paving a path for dendrite penetration [299]. In rigid inorganic SSEs prone to crack propagation, it’s essential to regulate the magnitude of overpotential and distribute local current density during battery charging, below a length-dependent threshold for terminal dendrite crack propagation. That makes Li wetting treatment a key aspect, equivalent to relaxing requirement on the SSE fracture toughness.

The rigidity of inorganic SSEs hampers their ability to effectively mitigate volume changes through deformation during cycling. Meanwhile, unavoidable heterogeneities in their structure and chemical composition will inevitably result in abundant defects at interfaces or along the grain boundaries, which can be easily triggered by even minor stresses. This exacerbates stress concentration inside the SSEs adjacent to LMA. Therefore, complete avoidance of crack propagation and dendrite propagation in hard ceramics is unattainable under such electrochemical-mechanical coupling mechanism. To address this critical issue, it is imperative to enhance the fracture toughness of bulk SSEs, improve interfacial wettability for better dispersion of local current density, for example, the direct utilization of high-flexibility composite polymer electrolytes.

## Design Strategies for LMB in Solid Electrolytes

To overcome the above-mentioned critical issues for ASSLMBs, various strategies have been put forward, they can be basically categorized by the following five kinds: interface engineering, fillers and additives, establishment of Li-ion conductive anode structure, alloyed anodes, and external field regulation. Of note, SSEs can completely replace the functions of separator, and other strategies shows similarities to those in liquid electrolytes. While, with the changing of physical and chemical properties, there exists much variation in LMBs when using SSEs.

### Interface Engineering

**Fig. 13:**
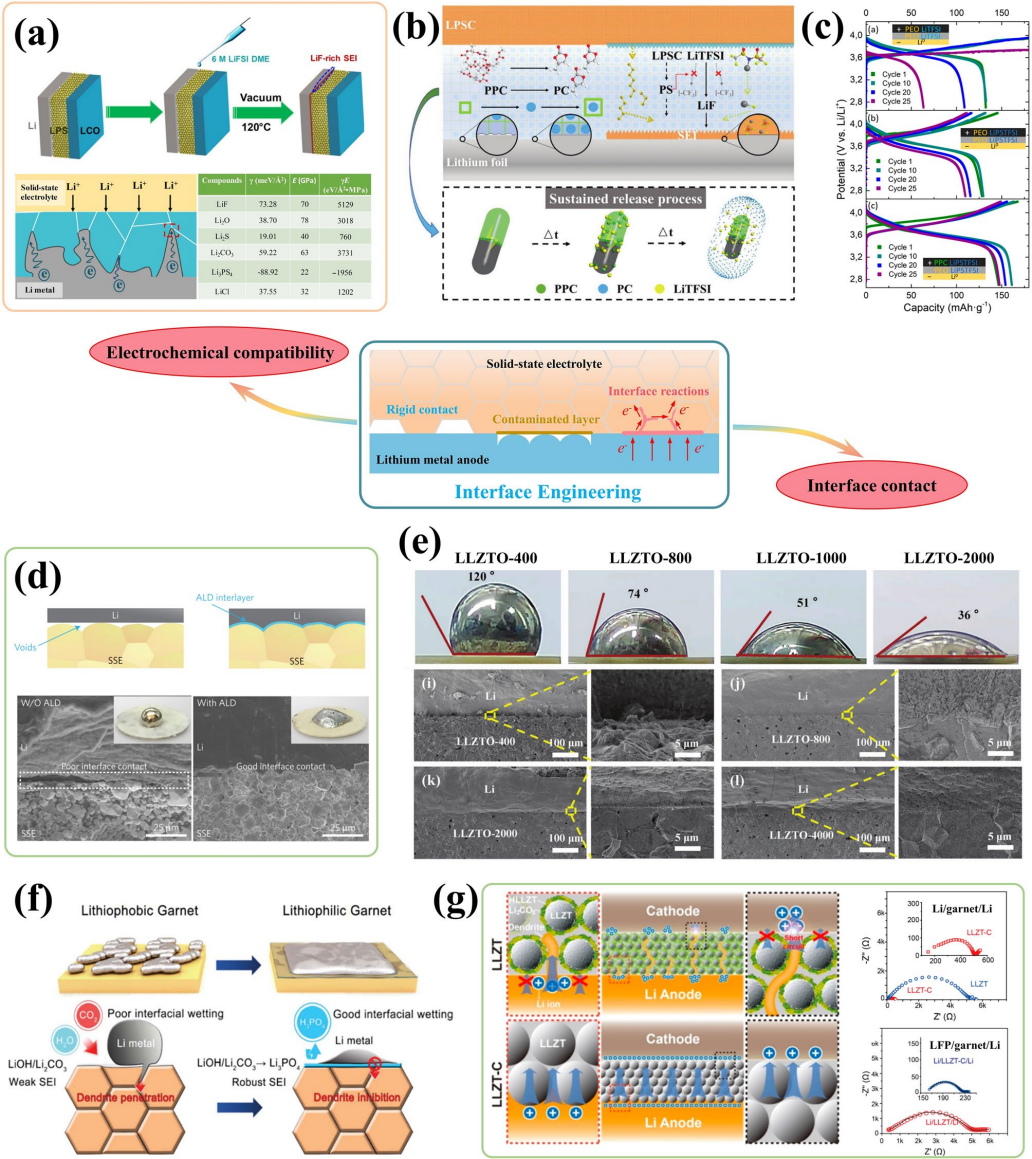
Interface engineering for LMA in ASSLMBs. **a** Schematic illustration of the formation of LiF-rich SEI layer between LPS electrolyte and LMA, and the corresponding evaluation from the perspective of critical Li dendrite length. Reproduced with permission from [302] Copyright 2018, The American Association for the Advancement of Science. **b** Schematics of sustained release effect of PPC between LPSC electrolyte and LMA. Reproduced with permission from [306] Copyright 2020, Wiley–VCH. **c** Voltage profiles for PEO-PPC double-layer electrolyte in NCM622/Li cell. Reproduced with permission from [307] Copyright 2022, American Chemical Society. **d** Schematics of the wetting behavior of garnet surface with molten Li after Al2O3 coating. Reproduced with permission from [315] Copyright 2016, The Authors, published by Springer Nature. **e** Wetting behaviors to molten Li of a series of different surface morphologies LLZTO pellets obtained by the grinding of 400, 800, 2000 and 4000 grit sandpapers. Reproduced with permission from [318] Copyright 2023, Wiley–VCH. **f** Schematic illustration of the formation process and roles of the Li3PO4 modification layer between the garnet SSE and LMA. Reproduced with permission [320]. Copyright 2019, Royal Society of Chemistry. **g** Schematic illustration of ASSLMBs with garnet LLZT and carbon-treated LLZT and EIS spectra for Li/garnet/Li and LFP/garnet/Li cells. Reproduced with permission from [321] Copyright 2018, American Chemical Society

The interface engineering can effectively restrict parasitic reactions resulting from the incompatibility between SSEs and LMA, as well as significantly enhance interface contact. It has been reported that an ultrathin coating layer of BN, ZnO, LiPON is effective in blocking interface reaction with LATP, LAGP and sulfide electrolytes, and maintaining low and stable interface resistance during cycles [258, 259, 300, 301]. Su et al. introduced a thin amorphous LiPON interlayer between argyrodite Li_6_PS_5_Cl electrolyte and LMA to facilitate rapid lithium-ion transport across the interface and suppress side reactions due to the decent ionic conductivity and electronic insulating properties of LiPON [259]. Consequently, the interfacial resistance is thus reduced to a mere 1.3 Ω cm^2^, enabling stable Li plating/stripping cycling for over 1000 h with a stabilized polarization voltage of 7.9 mV under a current density of 0.5 mA cm^−2^. By utilizing the reaction of 6 M LiFSI-DME highly concentrated electrolyte on LMA, Fan and co-workers successfully construct an in-situ LiF-rich interface towards Li_3_PS_4_ electrolyte (Fig. 13a) [302]. On account of extremely low electronic conductivity and the inherent electrochemical stability of LiF, it elevates the critical current density (CCD) of Li_3_PS_4_ (LPS) to > 2 mA cm^−2^ and achieves high Coulombic efficiency of over 98% for Li plating/stripping. This indicates improved endurance to current density and suppression of Li dendrite growth during cycling without cell failure. The ability of interface to suppress Li dendrite growth was evaluated by the critical Li dendrite length before the dendrite grows (${L}_{c}$), which is defined by Eq. (7):

7 $$L_{c} = \frac{2\gamma E}{{\pi \sigma^{2} }}$$

$\gamma$ denotes the interfacial energy required to form a new interface towards LMA per unit surface area. $\sigma$ is the stress at the crack tip or grain boundaries. E represents the bulk modulus. It has been estimated that spontaneous growth can occur once the calculated dendrite length exceeds ${L}_{c}$,. Therefore, a higher value of ${L}_{c}$ indicates greater suppression ability against Li dendrites, and $\gamma E$ represents the intrinsic property of SSEs or interface to withstand the growth Li dendrites. LPS exhibits a negative interfacial energy (− 88.92 meV Å^−2^), indicating its inherent instability when attaching with LMAs. In comparison with other normal interface components such as Li_2_O, Li_2_S, Li_2_CO_3_ and LiCl, LiF shows the highest interfacial energy (73.28 meV Å^−2^) and fine bulk modulus (70 GPa), resulting in the highest value of $\gamma E$ (5129 eV Å^−2^ MPa). That makes LiF-rich interface an exceptional electron blocking layer for sulfide SSE. Furthermore, Ji et al. designed a cold-pressed Li_3_N-LiF composite interlayer against LPS electrolyte [303]. Such an interlayer combines both the merits of LiF and Li_3_N, demonstrating high ionic conductivity, low electronic conductivity, and strong interface energy. This can further enhance CCD to > 6 mA cm^−2^ and achieve a Coulombic efficiency of 99% after 150 cycles.

Soft polymer electrolytes, acting as an interfacial buffer layer, can establish intimate contact at the interface while inhibiting side reactions, thereby significantly reducing the internal resistance of cells. For instance, a sticky PEO thin layer reduces the interfacial impedance of symmetric Li/LATP/Li cell by nearly one order of magnitude [304]. Zhou et al. synthesized a composite polymer comprising PEO and cross-linked poly(ethylene glycol) methylether acrylate as a coating layer on garnet pellets, which exhibits an approximate Li-ion transference number of 0.9 [305]. This also contributes to improved Li-ion transport and enhanced inhibition of Li dendrites growth. Wen’s group introduced a PPC-LiTFSI layer to prevent the direct contact between LPSC and LMA while swelling the coating layer for full contact with LMA through gradual degradation of PPC (Fig. 13b) [306]. Meanwhile, through the sustained release of polysulfides, P_2_S_7_^4−^ and P_2_S_6_^4−^ species, an ultra-stable SEI composed of LiF and PS_4_^3−^ is induced, resulting in homogeneous Li deposition. Consequently, symmetric Li/Li cells exhibit stable cycling for 1200 h at 0.1 mA cm^−2^ and even 300 h at 0.5 mA cm^−2^ without any short circuit occurrences. Taking advantage of the varying electrochemical stability among different electrolytes, Aranguren et al. [307] proposed a PEO-PPC double-layer polymer electrolyte, respectively improving LMA and high-voltage stability. Using lithium poly(4-styrenesulfonyl (trifluoromethylsulfonyl)imide) (LiPSTFSI) in place of LiTFSI as Li salts, the Li-ion transference number has been elevated from 0.32 to 0.98, which leads to smooth cycling for symmetric Li/Li. Specifically, NCM622/Li cells with this PEO-PPC double-layer electrolyte demonstrate higher capacity delivery as shown in Fig. 13c.

Enhancing the interface contact performance in garnet-type SSEs with lithium anode is crucial, and can be achieved by improving wettability through full swelling of the rigid surface using molten Li. To reduce interfacial impedance and accelerate Li-ion transport, various thin deposited layers such as Au [308], Ag [309], SiO_2_ [310], Cu_3_N [311], SnN_x_ [312] have been employed to construct a mixed electron/ion conductive interface via in-situ reactions at the surface [313, 314]. It is worth noting that interfacial impedance between garnet-type Li_7_La_2.75_Ca_0.25_Zr_1.75_Nb_0.25_O_12_ (LLCZN) and LMA can be remarkably reduced from 1710 to 1 Ω cm^2^ at RT using an ultrathin Al_2_O_3_ coating layer via ALD [315]. That oxide layer enables good wetting of metallic Li and allows effective Li-ion transport (Fig. 13d), thereby contributing to stable cycling coupled with Li_2_FeMn_3_O_8_ cathode. With the advancement of research, it has gradually been recognized that the contaminated Li_2_CO_3_ layer resulting from slight garnet decomposition upon exposure to air is the fundamental cause of poor wettability and high interface impedance against LMA [316, 317]. Therefore, it has been confirmed as effective ways to polishing the Li_6.5_La_3_Zr_1.5_ T a_0.5_O_12_ (LLZTO) pellet by sandpapers to acquire low-roughness surface and treat by acids to remove Li_2_CO_3_ contaminated layer [318–320]. In Liang’s research, different surface morphologies were obtained using 400, 800, 2000 and 4000 grit sandpapers with root mean square roughness of 114, 107, 40.8, and 20.6 nm respectively [318]. As the grinding became finer and the roughness decreased, closer interface contact was achieved as evidenced by lower wetting angles of 120°, 74°, 51° and 36° (Fig. 13e). After acid washing of H_3_PO4, the Li_2_CO_3_/LiOH passivation layer is eliminated and replaced by a uniform Li_3_PO_4_ modification layer, which enhances interfacial wettability and serves as a stable interface to prevent Li dendrite penetration. As a result, it exhibits significantly reduced interfacial resistance of 7.0 Ω cm^2^ and stable galvanostatic cycling for over 450 h at 0.5 mA cm^−2^ in symmetric Li/Li cells (Fig. 13f). Furthermore, Goodenough’s group discovered that the surface of carbon-treated garnet pellets is free of Li_2_CO_3_ after annealing at 700 °C [321]. Simultaneously, the secondary Li−Al−O glass phase with low Li-ion conductivity in the grain boundary was also minimized (Fig. 13g). This highlights the effectiveness of carbon post-treatment in reducing interfacial resistances to 28 and 92 Ω cm^2^ for Li/garnet and garnet/LFP interfaces. Additionally, introducing Li_3_AlF_6_ during sintering process can similarly enhance interface contact of stiff garnet pellets and LMA [322].

### Fillers and Additives

#### Selection of Fillers

To reconcile the inherent contradiction between ionic conductivity and electrochemical stability, as well as to achieve a balance between high mechanical strength and intimate contact, solid composite electrolytes (SCEs) comprising a polymer matrix and inorganic particles as secondary phase offer a promising avenue for practical ASSLMBs [323]. In polymer electrolytes, Li-ion transport is governed by the dissociation of Li salts and subsequent movement of Li^+^, particularly within amorphous regions. The incorporation of second-phase particles can lower the glass transition temperature (T_g_) and disrupt the initial high crystallinity of the polymer matrix, ultimately enhancing the movement of chain segments [324]. Furthermore, the utilization of Lewis acid–base fillers can further augment the dissociation of Li salts, thereby elevating the concentration of mobile Li^+^ in bulk electrolyte and significantly enhancing the ionic conductivity [253, 325]. By combining the high flexibility of polymers with the superior mechanical strength derived from inorganic ceramic fillers, SCEs effectively address the trade-off between intimate interface contact and necessary rigidity to withstand Li dendrite growth [326]. Besides, the exceptional processability makes SCEs the few SSEs that are suitable to large-scale manufactures. In some aspects, the thermal stability of SCEs can be enhanced after incorporating inorganic fillers. The thermally stable skeleton structure of these fillers ensures that the cathode and anode remain separated even if the polymer components decompose or burn at high temperatures [327]. In some cases, inorganic fillers can function to stabilize the interface and contribute to the broaden electrochemical window [328, 329]. Therefore, careful selection of appropriate inorganic fillers is crucial for fabricating SCEs.

**Fig. 14:**
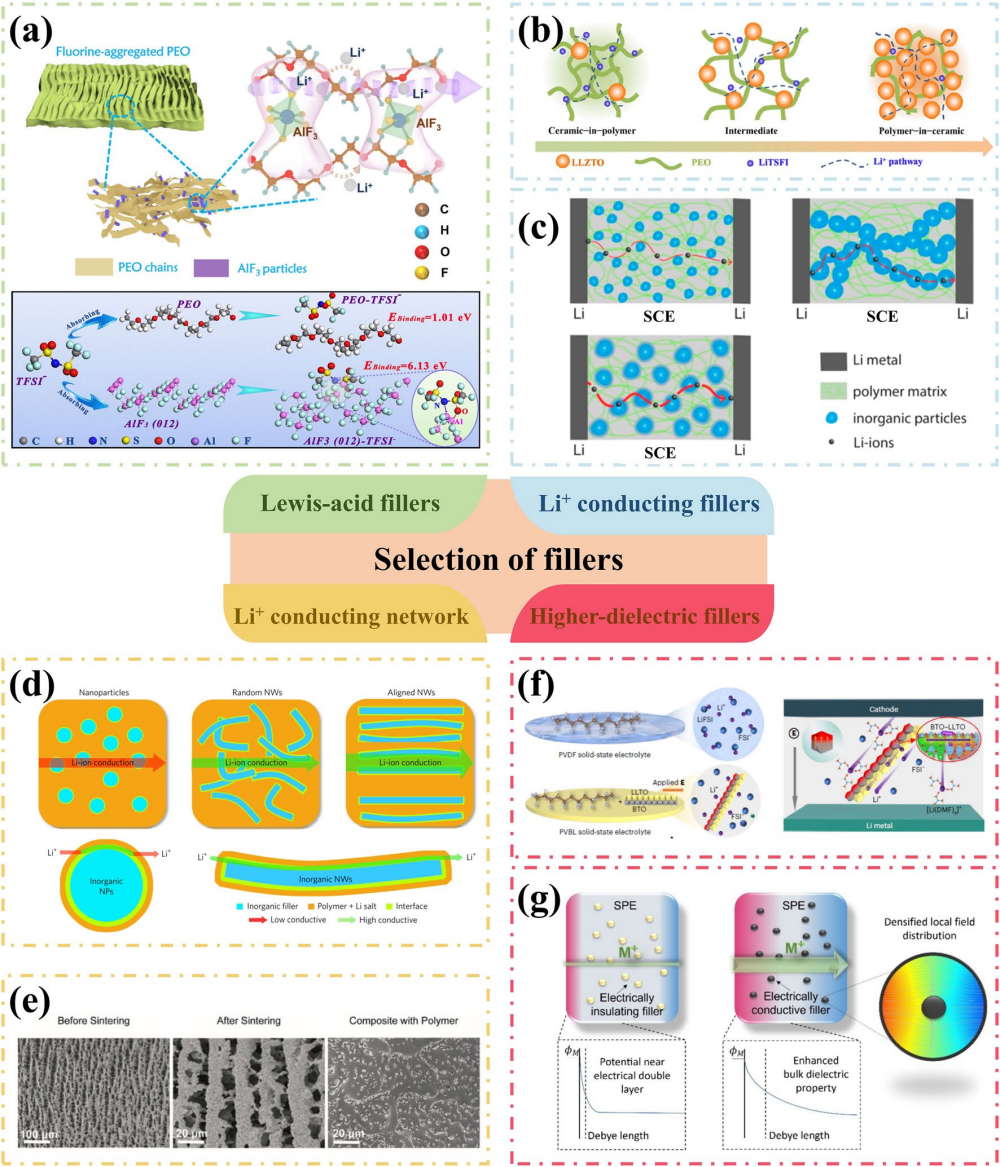
Selection of fillers for solid composite electrolytes (SCEs). **a** Schematic illustration of fluorinated PEO-based electrolyte by Lewis-acid AlF3 filler. Reproduced with permission from [338] Copyright 2022, The Authors, published by Springer Nature. **b** The selection of Li-ion conducting fillers and the varying performance under different content of LLZTO nanoparticles. Reproduced with permission from [327] Copyright 2018, Elsevier. **c** Schematic illustration of Li-ion diffusion pathways in PEO-based SCEs with different amount of nanofillers. Reproduced with permission from [341] Copyright 2022, American Chemical Society. **d** Li-ion conduction pathways in SCEs with nanoparticles, random nanowires and aligned nanowires. Reproduced with permission from [343] Copyright 2017, The Authors, published by Springer Nature. **e** SEM images of ice-templated LAGP framework. Reproduced with permission from [345] Copyright 2019, Elsevier. **f** Schematic illustration of the Li salt state in SCE composed of PVDF matrix and BaTiO3-LLTO nanowires coupling the ceramic dielectric and inorganic SSE. Reproduced with permission from [349] Copyright 2023, The Authors, published by Springer Nature. **g** Schematics illustration of the electric field distribution of conductive filler in PEO electrolyte. Reproduced with permission from [350] Copyright 2023, Wiley–VCH

Inert oxide particles such as Al_2_O_3_ [330], LiAlO_2_ [331], SiO_2_ [332], and TiO_2_ [333] are initially selected as fillers for PEO-based electrolytes to reduce crystallization and enhance the mobility of chain segments. It has been discovered that particle size, volume fraction, and associated chemical groups significantly influence electrochemical behaviors of SCEs [249]. The interface between inorganic fillers and polymer matrix plays a crucial role in Li-ion conduction [334, 335]. So, it will acquire better ionic conductivity with larger interface region resulted from reduced particles size. For example, when the Al_2_O_3_ particle size is reduced from 10 μm to 10–20 nm, the ionic conductivity of PEO-Al_2_O_3_ SCE increases by nearly one order of magnitude [336]. It has been observed that the maximum ionic conductivity occurs within a volume fraction range of fillers between 5 and 20% [337]. However, exceeding this critical value results in a decline in ionic conductivity due to ion channels blockage, despite the continued enhancement in mechanical strength. To address this issue, Hu et al. fabricated a fluorinated SCE by introducing mesoporous α-aluminum fluoride with high specific surface area (HS-AlF_3_) into PEO matrix [338]. This HS-AlF_3_ exhibits pronounced Lewis acidity, which promotes the dissociation of adjacent LiTFSI and enhances the overall of Li^+^ transport, contributing to an elevated Li-ion transference number of up to 0.67. Besides, density functional theory (DFT) calculations confirm the potent adsorption effect of AlF_3_ surface on TFSI^−^ anion (Fig. 14a). What’s more, well-dispersed fillers with high F-content can create a smooth interface concentrated in F and promote the enrichment of Li_2_O phase at the interface. As a result, optimized symmetric Li/Li cells exhibit relatively low interfacial resistance and overpotential (25, 50, and 75 mV for 0.1, 0.2, and 0.4 mA cm^−2^) during long-term cycling. The laminated all-solid-state FeF_3_/Li cells exhibit an initial specific discharge capacity of approximately 600 mAh g^−1^ at a current density of 700 mA g^−1^ and maintain a capacity of 200 mAh g^−1^ after 900 cycles at 60 °C. Due to the negatively charged surfaces, natural nano-clay halloysite and layered vermiculite sheets are incorporated into PEO to facilitate the separation of lithium salts into Li-ions, enabling rapid Li-ion transport through the bulk SSEs [326, 339].

Incorporating nanoscale ceramic Li-ion conducting particles, such as LLZO, LLTO, LATP, LAGP and others, is advantageous in enhancing the overall electrochemical properties of SCEs [340]. Chen et al. prepared SCEs based on PEO, with varying content of LLZTO from 10 to 80%, and investigated their electrochemical performance during the transition from a “ceramic in polymer” system dominated by PEO to a “polymer in ceramic” system dominated by inorganic particles (Fig. 14b) [327]. These composite electrolytes exhibit enhanced ionic conductivity (> 10^−4^ S cm^−1^ at 55 °C) and stable antioxidant potential, facilitating excellent cycling performance of all-solid-state LFP/Li batteries. As the LLZTO content increases, the Li^+^ transport pathways gradually shift from the PEO phase to the loosely connected network structure of LLZTO particles (Fig. 14c) [341]. The SCE with PEO as its main component exhibits superior flexibility and interfacial contact performance while incorporating LLZTO ceramic particles as the primary constituent demonstrates higher mechanical strength and enhanced safety tailored for specific application scenarios.

To establish a continuous network of inorganic materials that conduct Li-ions is a crucial step towards enhancing ionic conductivity [342]. According to the research of Cui’s group, SCEs with well-aligned inorganic Li-ion conductive nanowires exhibits much higher ionic conductivity (6.05 × 10^−5^ S cm^−1^ at 30 °C), which is nearly one order of magnitude enhancement compared to random oriented nanowires [343]. This can be attributed to the fast ion-conducting pathways without crossing junctions on the surfaces of the aligned nanowires (Fig. 14d). Wan et al. utilized the electrospinning to synthesize 3D cross-linked continuous nanowires of LLZO with diameters ranging from 100 to 200 nm, and then fused this network into PEO-LiTFSI matrix through direct immersion to ultimately fabricate an SCE [344]. Except for the presence of fast Li^+^ transport channels along LLZO networks, the significantly enhanced mechanical strength of SCE induces  uniform deposition of metallic Li and effectively inhibits the growth of Li dendrites, demonstrating stable cycling in symmetric Li/Li cells for 1000 h at 60 °C without short circuits and in LFP/Li cells both at 60 and 45 °C. Furthermore, Wang et al. successfully employed the ice template method to fabricate vertical arrays of LAGP for PEO/PEG polymer electrolytes, which facilitates efficient Li-ion transport through direct pathways (Fig. 14e) [345]. Consequently, the resulting electrolyte exhibits an impressive ionic conductivity of 1.67 × 10^−4^ S cm^−1^ at RT, nearly 6.9 times over that with dispersed LAGP nanoparticles.

Ferroelectric ceramic particles, such as BaTiO_3_, PbTiO_3_, and LiNbO_3_, have been utilized as fillers for the fabrication of high-performance SCEs [346, 347]. This is attributed to their significantly higher dielectric constant (ε_r_) and resulting permanent dipole moment which can facilitate the dissociation of LiTFSI into a greater number of free charge carriers and enhance their mobility [254]. Specifically, He’s group conducted a comparative study on the electrochemical performance of poly(vinylidene fluoride-co-trifluoroethylene-co-chlorotrifluoroethylene) [P(VDF-TrFE-CTFE)], a relaxor ferroelectric polymer, and traditional PVDF electrolyte [348]. With an increased ε_r_ value from 9 to 44, P(VDF-TrFE-CTFE) polymer electrolyte exhibits substantially higher ionic conductivity of 3.10 × 10^−4^ S cm^−1^ at RT and lower activation energy of 0.26 eV compared to PVDF electrolyte. The latter only demonstrates a conductivity of 1.77 × 10^−5^ S cm^−1^ and an activation energy of 0.49 eV. Therefore, the hybrid PVDF electrolyte blended with the P(VDF-TrFE-CTFE) or ferroelectric ceramic fillers such as BaTiO_3_ displays enhanced ionic conductivity and reduced polarization, enabling stable cycling for up to 1,200 and 1,650 h without short circuits at 25 and 45 °C respectively without any short circuits. Additionally, He’s group proposed a SCE consisting of PVDF matrix and BaTiO_3_-LLTO nanowires with a side-by-side heterojunction structure that couples the ceramic dielectric and inorganic SSE (Fig. 14f) [349]. These synergetic effects contribute to high ionic conductivity of 8.2 × 10^−4^ S cm^−1^ and Li-ion transference number of 0.57 at 25 °C while homogenizing the interfacial electric field with electrodes, ultimately enabling over 1500 cycles at a current density of 180 mA g^−1^ in NCM811/Li cells.

The ideal electrolyte should possess excellent ionic conductivity while exhibiting absolute electron blocking capability, as excessive electron conductance can result in self-discharge and exacerbate undesired side reactions at the interface. However, recent studies have indicated that the impact of conductive fillers on SCEs is intricate, and a small amount of introduction without the formation of continuous electronic pathways may aid in reducing overpotentials and enhance overall electrochemical performance. Guo et al. have evaluated the advantages and limitations of electronically conductive domains in solid polymer electrolytes [350]. On a macroscopic scale, the carbon filler serves to densify the local electric field distribution and facilitate charge transfer, thereby impeding Li dendrite formation at particle boundaries (Fig. 14g). On a macroscopic scale, incorporating carbon filler enhances the dielectric properties of PEO matrix, promoting solvation and diffusion processes of Li salts while homogenizing Li-ion concentration distribution and reducing potential for Li^+^ depletion. Therefore, deliberate incorporation of 1 wt% Super P enables stable cycling life, low overpotential, and promising full cell performance in regular solid polymer electrolyte. Although there is ongoing debate regarding the selection of conductive fillers, this study underscores the significance of enhancing dielectric properties in SCEs.

#### Structure Design of Solid Composite Electrolytes (SCEs)

The selection of fillers may alter the electrochemical window and result in selective electrochemical stability of SCEs towards either the cathode or anode. Wang et al. designed a double-layer SCEs composed of two layers: PEO-LiTFSI and PEO-LAGP-LiTFSI [351]. The incorporation of LAGP enhances the stability of the PEO-based electrolyte at high voltages, while the PEO-LiTFSI layer attached to LMA prevents severe interface reactions between LAGP and metallic Li. Such solid electrolyte extends the electrochemical window to 5.12 V (vs. Li/Li^+^), enabling high-voltage LiMn_0.8_Fe_0.2_PO_4_ (LMFP) cells with good cycling stability to deliver an initial capacity of 160.8 mAh g^−1^. Moreover, Liu et al. [352] fabricated a double-layer SCE with a content gradient of inorganic LGPS. The LGPS-rich side, directly attached to the LMA, exhibits sufficient mechanical strength with a modulus of 6.67 GPa, acting as a protective layer against Li dendrites penetration. Meanwhile, the elastic side rich in polymer, with a modulus of only 0.25 GPa, is suitable for fully wetting porous electrodes and accommodating high-load cathode applications.

Additionally, the fabrication of ultra-thin SCEs is a crucial strategy to compensate the shortcomings in Li-ion transport associated with polymer-based electrolytes, thereby reducing overall internal resistance and enabling normal operations at lower temperatures. Chen et al. employed a tape casting method to fabricate a thin-film electrolyte with a thickness < 10 μm, comprising a high mass fraction (94.3 wt%) of PAN-nanocoated LLZTO powder and coated with a soft PEO buffer layer in contact with LMA [353]. The synthesized SCE not only possesses a Young’s modulus of 9.7 GPa, which effectively suppresses the growth of Li dendrites but also reduces the areal specific resistance (ASR) to 9 Ω cm^2^, signifying improved interfacial contact and fast Li-ion migration. He et al. proposed an ultrathin bilayer SCE with porous vermiculite skeleton and double-layer PEO/PAN polymer matrix [354]. The incorporation of rigid ceramic fillers enhances mechanical strength and thermal safety while the soft part of the bilayer polymer electrolyte stabilizes both LMA and high-voltage cathodes simultaneously. It is worth noting that the as-prepared SCE has a thickness of only 4.2 μm, which significantly reduces the transport distance of Li^+^. Consequently, all-solid-state batteries based on NCM811 demonstrate exceptional long-term stability exceeding 3000 h at an N/P ratio of 1 while achieving high energy densities of 506 Wh kg^−1^ and 1514 Wh L^−1^.

#### Effect of Additives

**Fig. 15:**
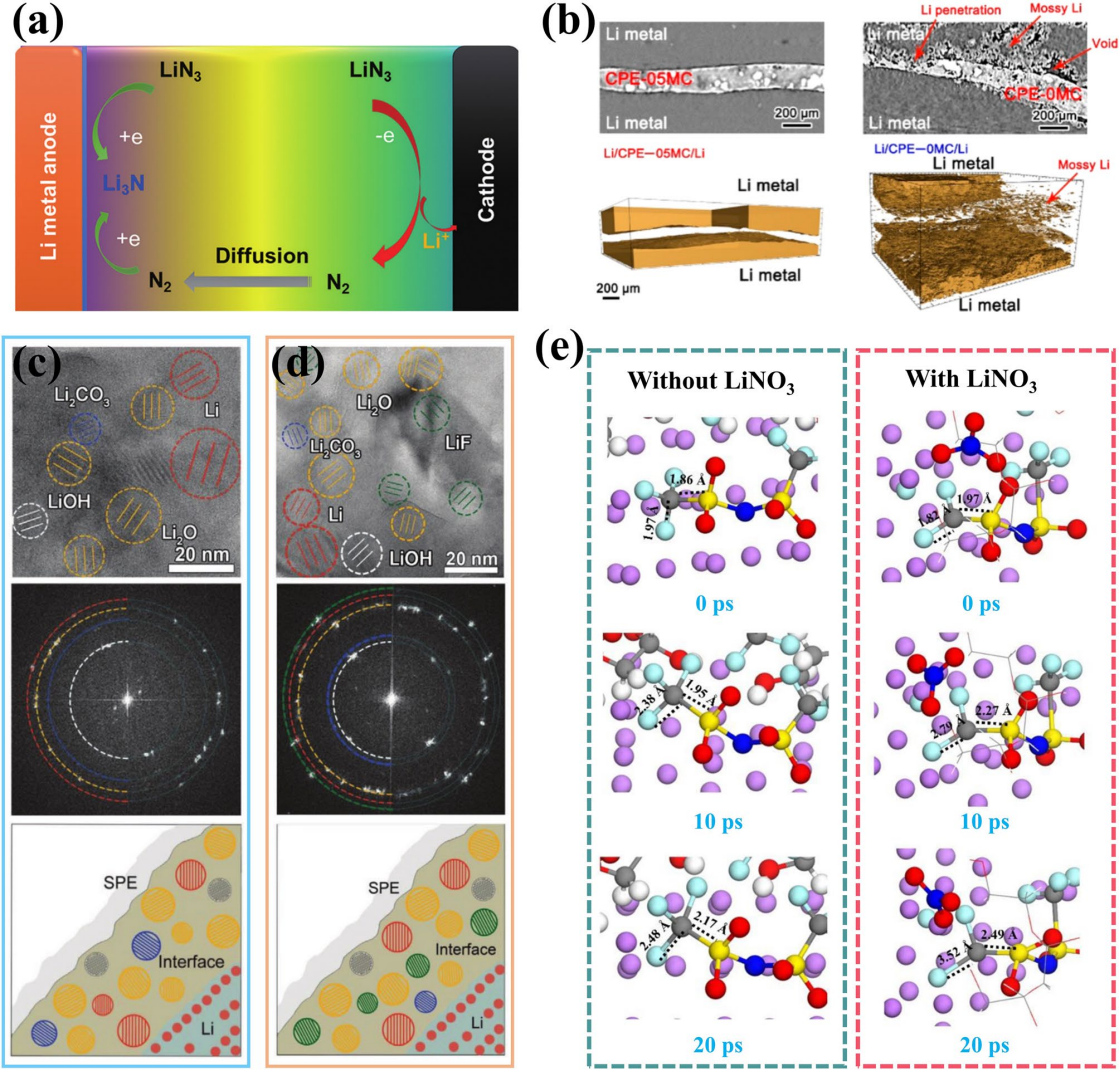
Effect of additives for solid composite electrolytes (SCEs). **a** Electrochemical reaction evolutions of LiN3 in ASSLMBs. Reproduced with permission from [358] Copyright 2017, Wiley–VCH. **b** Cross-sectional synchrotron X-ray tomography images of symmetric Li/Li cells with and without Mg(ClO4)2 additive. Reproduced with permission from [360] Copyright 2021, American Chemical Society. Cryo-TEM characterization of PEO/Li interface using **c** PEO-LiTFSI electrolyte and **d** PEO-LiTFSI-Li2S electrolyte. Reproduced with permission from [361] Copyright 2020, Wiley–VCH. [361]. **e** Ab initio molecular dynamics (AIMD) simulations of LiTFSI structural configuration changes without and with LiNO3 addition at 333 K adjacent to LMA surface. Reproduced with permission from [362] Copyright 2021, Elsevier

Similar to organic liquid electrolytes, additives also play an important role in polymer-based SCEs. For example, Yang et al. have verified the same self-healing electrostatic shield by introducing 0.05 M Cs^+^ to guide uniform Li deposition in PEO matrix [355]. Despite of the relative stability towards LMA, it still exhibits a strong tendency of PEO to decompose C_2_H_4_, H_2_ and Li_2_O over time. This decomposition gradually deteriorates the intimate contact at the PEO/Li interface and exacerbates uneven Li deposition [356, 357]. That makes a preferable interface an indispensable part for forming high-performance ASSLMBs, just like the regulation of SEI layer in liquid LMBs, which should be rich in stable inorganic chemicals, sufficient mechanical strength, fast Li-ion transport and charge-transfer kinetics. Armand et al. proposed an innovative electrolyte additive of lithium azide (LiN_3_) to generate a compact and highly conductive passivation layer on the LMA surface [358]. Except for the direct reduction on the surface of metallic Li, oxidation of LiN_3_ occurs at the cathode side leading to molecular N_2_ formation that subsequently migrates towards the anode side and generates additional Li_3_N (Fig. 15a). This mechanism eventually leads to the formation of a robust passivated interface with 2 wt% LiN_3_ addition, effectively inhibiting Li dendrites growth and polysulfides shuttling effect. Additionally, the introduction of 5 wt% black phosphorus can result in a Li_3_P layer at the interface, increasing the wettability of SCE to LMA, homogenizing the current density distribution to allow for doubled CCD value for symmetric Li/Li cells [359]. Goodenough’s team proposed Mg(ClO_4_)_2_ as an effective additive for PEO-based SCE [360]. Through a combination of experimental and computational studies, it has been demonstrated that the immobile Mg^2+^ ions can coordinate with ether oxygen and Li salt anions, thereby enhancing Li^+^ ion mobility in bulk phase. Simultaneously, an in-situ induced Li_2_MgCl_4_/LiF interface layer can homogenize the Li^+^ flux in the vicinity of LMA while increasing the CCD to a record of 2 mA cm^−2^. Cross-sectional synchrotron X-ray tomography images shown in Fig. 15b depict favorable interface contact and uniform deposition of lithium without mossy accumulation or penetration by dendritic structures when using symmetric Li/Li cells with 0.5 wt% Mg(ClO_4_)_2_ additive (referred to as CPE-05MC).

Furthermore, Sheng et al. have successfully modified the PEO/Li interface by introducing a Li_2_S additive and demonstrated its effect on prompting TFSI^−^ decomposition [361]. Cryo-transmission electron microscopy (cryo-TEM) images reveal the presence of abundant LiF nanocrystals within the mosaic structure, providing strong evidence for the formation of a LiF-enriched interface, as displayed in Fig. 15c, d. The same phenomenon can be achieved with the addition of LiNO_3_. Zhang et al. confirmed through Ab initio molecular dynamics (AIMD) simulations that the stretching of C-S and C-F bonds in TFSI^−^ anions induced by LiNO_3_ additive accelerates the decomposition of LITFSI to form LiF (Fig. 15e) [362]. Accompanied by the simultaneous reduction of LiNO_3_ on the LMA surface, it ultimately generates a Li_3_N-LiF-enriched interlayer, which can significantly reduce interfacial resistance and facilitate homogeneous Li deposition. A small amount of LiPO_2_F_2_ not only stabilizes the LMA through as-formed protective layer consisting of LiF and Li-P species, but also extends the oxidation voltage of PEO-based SCE to over 4.5 V by actively decomposing at the cathode side [363]. Therefore, with moderate LiPO_2_F_2_ addition, superior cycling performance is demonstrated as it retains 94.9% of initial discharge capacity after 800 cycles at 1 C, enabling stable cycling behavior in assembled NCM622/Li cells with a specific capacity of 147.8 mAh g^−1^ and an average coulombic efficiency of 99.53% after 100 cycles. In addition, other additives such as Li_2_S_6_ [364], I_2_ [365], reactive Li salts [366] and Si nanoparticles [367] have been successively utilized as additives, to supplement the electrochemical performance deficiencies in SCEs.

### Li-ion Conductive Anode Construction

**Fig. 16:**
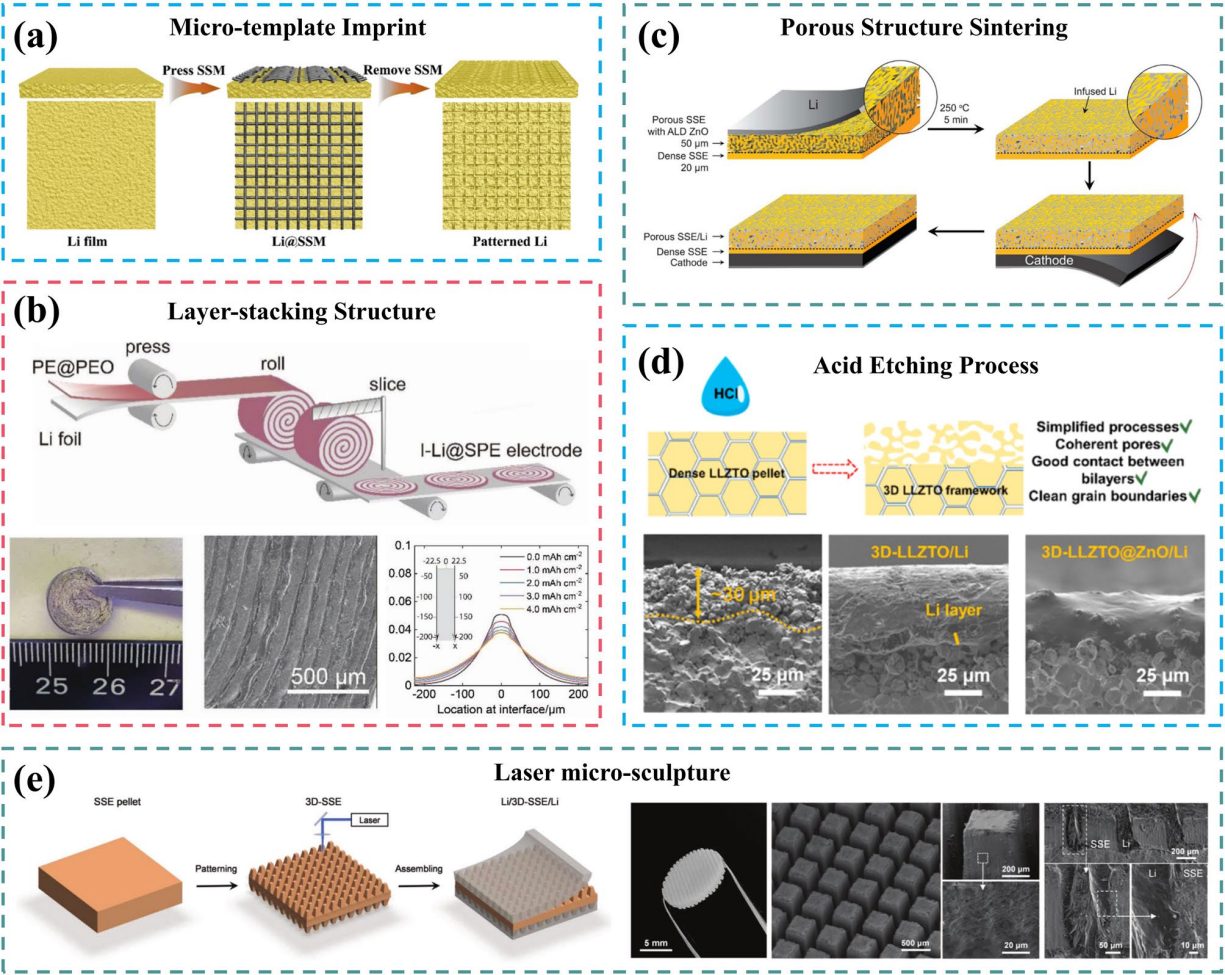
Li-ion conductive anode structure design for LMA in ASSLMBs. **a** Schematic illustration of patterned LMA fabricated by micro-template imprint method. Reproduced with permission from [368] Copyright 2020, Elsevier. **b** Schematic for the manufacturing process, SEM images and COMSOL simulation for a layered-stacking anode structure. Reproduced with permission from [369] Copyright 2022, Wiley–VCH. **c** Schematic illustration of sintering garnet porous structure. Reproduced with permission from [371] Copyright 2018, Elsevier. **d** Schematics and SEM characterizations of acid etching process on a garnet SSE by HCl. Reproduced with permission from [373] Copyright 2020, American Chemical Society. **e** Fabrication and characterization of 3D-SSE via laser micro-sculpture method. Reproduced with permission from [375] Copyright 2021, Wiley–VCH

Unlike liquid electrolytes, which can easily infiltrate into the 3D electron-conductive frameworks and conveniently establish Li-ion transport channels, it is imperative to manually construct Li-ion transport pathways within anode structures to better accommodate dramatic volume changes under high-loading cycles. The specific preparation method involves the treatment at LMA side or SSE side. Through a low-cost micro-template imprint method, it can obtain a 3D LMA structure composed of numerous square Li with deep grooves around 100 μm (Fig. 16a) [368]. These grooves are formed by directly pressing stainless-steel mesh onto the Li foil, facilitating the dispersion of concentrated current density and guiding preferential Li deposition inside, thereby suppressing the growth of Li dendrites. By repeatedly stacking thin Li foil and PEO layer, Cui’s team proposed an interdigitated Li-solid polymer electrolyte framework (I-Li@SPE) as the anode (Fig. 16b) [369]. In this manner, the conventional interface contacts between LMA and SSE have been transformed from planar to 3D, thereby promoting closer contact and expanding the active surface area for Li deposition. The COMSOL simulation results also indicates a reduction in local current densities of over 40% and a moderation of interfacial variation by over 50%. It is demonstrated that the CCD value has been increased from 0.4 to 1 mA cm^−2^ enabling high-capacity cycling of symmetric Li/Li cells can be achieved under 4 mAh cm^−2^. Moreover, Cui’s team proposed the incorporation of a flowable interfacial layer within the anode structure of metallic Li-reduced graphene oxide (Li-rGO) host to accommodate interfacial fluctuations and ensure superior adhesion to SSE [370]. Such flowable interfacial layer, composed of poly(ethylene glycol) (PEG) and LiTFSI, resembles a viscous semiliquid and can be impregnated into the Li-rGO host through thermal infiltration, furtherly offering Li-ion transport pathways inside. The specially designed anode structure is well-suited for high current density and mass loading conditions, enabling competitive rate performance in LFP/Li cells. It enables LFP/Li cells with respective capacity release of 141 and 110 mAh g^−1^ for 1 and 5 C, and 93.6% capacity retention after 300 cycles at 3 mA cm^−2^.

For SSE side, Hu et al. fabricated a porous-dense bilayer structured garnet SSE as a 3D ionic skeleton for LMA through sintering process (Fig. 16c) [371]. The porous layer functions as a stable ion-conducting host to accommodate migrated Li deposition, and the consequent large contact area reduces the local current density, producing lower overpotentials and more uniform distribution of deposited Li during cycling. The porous structure is also well-suited for the cathode, facilitating highly reversible cycling of ASSLMBs under large C-rate and high mass loading conditions [372]. When HCl is used to remove the Li_2_CO_3_-contaminated layer on the garnet surface, it gradually infiltrates into the bulk electrolyte along the grain boundaries, resulting in etching and formation of a porous Li-ion conductive framework in the surface region (Fig. 16d) [373]. By incorporating an ALD ZnO coating layer, the porous layer can establish intimate contact with molten Li and ultimately enhance CCD to 1.4 mA cm^−2^ in LLZO electrolyte. The facile construction of anode structure is attributed to the etching effect of strong acids [374]. Cui’s group has recently developed a novel 3D-micropatterned SSE using laser micro-sculpture method to stabilize the morphology of SSE/Li interface (Fig. 16e) [375]. The laser cutter with approximately 20 µm resolution enables fabrication of three-dimensional micropatterns with characteristic dimensions ranging from 200 to 400 µm. They also discussed optimizing the balance of high-energy laser beams to reduce processing time while minimizing mechanical damage and undesired chemical composition changes.

### Alloyed Anode

**Fig. 17:**
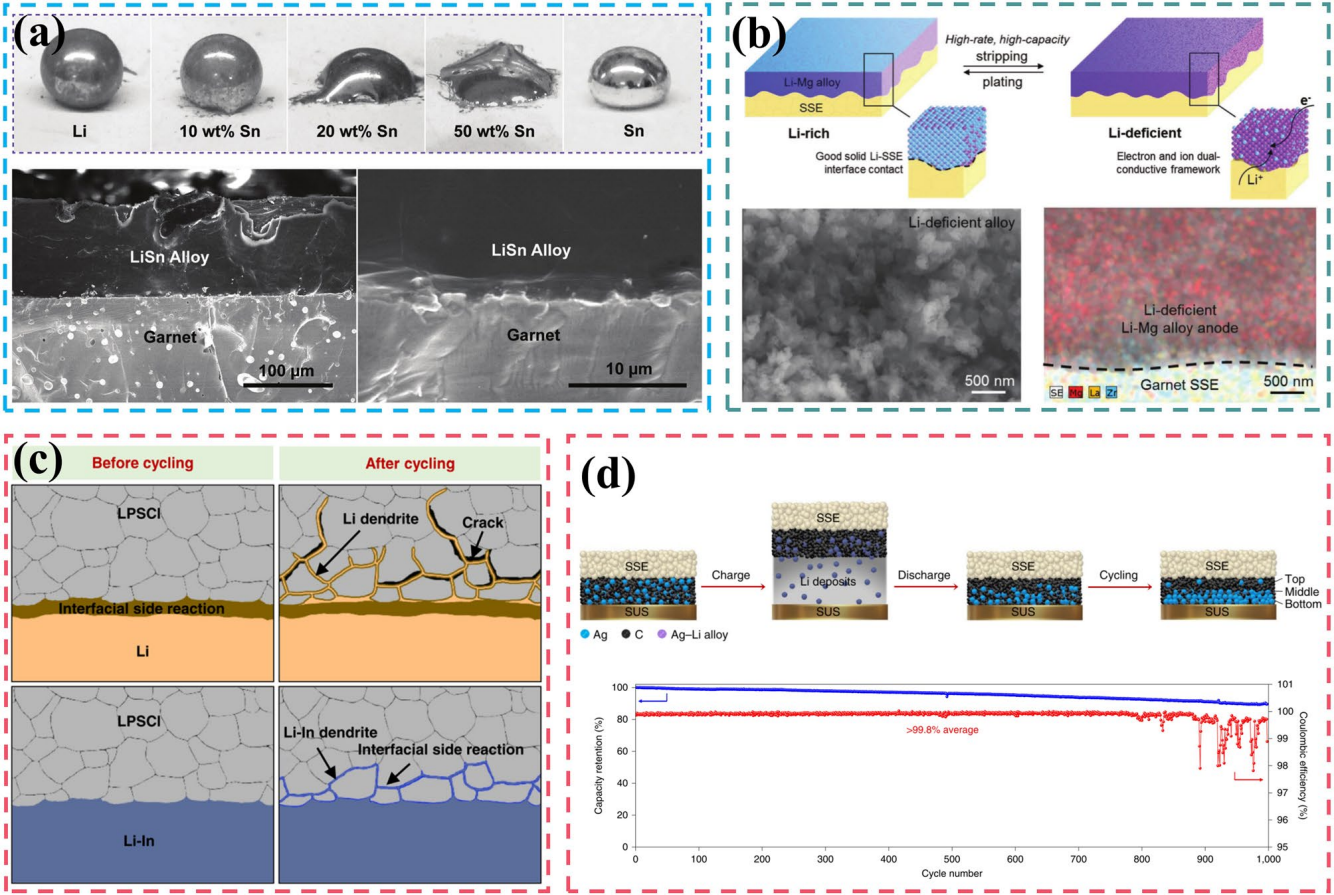
Alloyed anode design for LMA in ASSLMBs. **a** Wettability comparison of Li-Sn alloys on garnet substrates. Reproduced with permission from [376] Copyright 2017, Wiley–VCH. **b** Schematics and SEM image with elemental mapping within Li-Mg alloy anode during the Li stripping/plating process. Reproduced with permission from [377] Copyright 2018, Wiley–VCH. **c** Schematic illustration of Li dendrites propagation along the LPSC/Li and LPSC/Li-In interface. Reproduced with permission from [386] Copyright 2021, The Authors, published by Springer Nature. **d** Schematic illustration of Ag-C nanocomposite layer during Li stripping/plating process and corresponding electrochemical performance of NCM/Li cells. Reproduced with permission from [387] Copyright 2020, The Authors, published by Springer Nature

The primary function of alloyed anodes in oxide SSEs is to enhance interface contact, which is attributed to the increased surface energy and viscosity of molten Li upon introducing alloying elements, thereby improving its wettability on oxide SSEs. Wang et al. investigated the correlation between the ratio of alloy elements and molten Li wettability, revealing that binary alloys (Li-Sn, Li-Zn, Li-Si and Li-Al) exhibit enhanced wettability when the weight percentage of alloy elements exceeds 20 wt% (Fig. 17a) [376]. So that the molten Li-Sn alloy (30–50 wt% of Sn) ensures a tight and conformal contact with LMA, resulting in an interfacial resistance as low as 7 Ω cm^2^. Similar to their application in liquid electrolytes, the alloyed anodes also function as a stable electron/ion dual-conductive host structure. During the continuous Li plating/stripping process, the Li-Mg alloy undergoes repeated transformations between a Li-rich and a Li-deficient state [377]. This is confirmed by the time-of-flight secondary ion mass spectroscopy (ToF–SIMS) results on the cross-section of the alloy/SSE interface, which clearly demonstrate a distinct transition from highly concentrated to depletion of metallic Li (Fig. 17b). At the same time, there is no change in Mg intensity, indicating that the topological and microstructural features of the Li-Mg alloy framework can be effectively preserved even under a Li-deficient state. Besides, it has been proposed to use molten Li containing trace amounts of alloy elements (less than 1 wt%), such as Li-Na [378], Li-C [379], and Li-Sr [380], to address the issue of insufficient interface contact at the garnet/Li interface.

For sulfide SSEs, the utilization of alloyed anodes with relatively higher reduction potential and reduced chemical reactivity is primarily aimed at achieving better compatibility. Pan et al. proposed a Li_0.8_Al alloyed anode to pair with LGPS electrolyte demonstrating its exceptional electrochemical stability through steady cycling for over 2500 h at 0.5 mA cm^−2^ in symmetric cells[381]. When combined with an S cathode, the assembled Li–S full cell exhibits a high specific energy of 541 Wh kg^−1^ at a N/P ratio of 1.125, providing a viable alternative for constructing advanced ASSLMBs. Meng et al. reported a 99.9 wt% micro-silicon anode to pair with LPSC electrolyte [382]. This carbon-free high-loading silicon anode can effectively eliminate continuous interfacial side reactions by significantly reducing electronic conductivity, as compared to the mixed anode of micro-silicon and 20 wt% carbon additives. The stable cycling performance of all-solid-state NCM811/Li full cells is demonstrated, showing high-capacity retention of 80% after 500 cycles and an average Coulombic efficiency exceeding 99.9%, under conditions where the cathode loading is set at 25 mg cm^−2^ and current density is maintained at 5 mA cm^−2^.

In most cases, Li-In alloy is the most popular anode used in sulfide- and halide-SSEs batteries, for its balanced performance in theoretical capacity (1012 mAh cm^−2^), average potential (0.3 V vs. Li/Li^+^) [383], volume expansion (105%) and Li-ion diffusion rate (~ 10^−8^ cm^2^ s^−1^) [384, 385]. However, despite these advantages, alloyed anodes still face challenges in completely preventing the propagation of lithium dendrites. Luo et al. observed laterally striped Li-In dendritic structures within LPSC electrolyte that exhibit a more favorable morphology for reducing dendrite growth rate and mitigating structural damage to sulfide-SSEs compared to vertically growing lithium dendrites (Fig. 17c) [386]. Nevertheless, it remains crucial to inhibit interfacial side reactions in order to decelerate the propagation rate of Li-In dendrites and minimize the likelihood of SSE failure. Lee et al., on the other hand, an alternative approach by incorporating an Ag-C nanocomposite layer instead of using traditional lithium metal foil as the anode [387]. The inclusion of Ag nanoparticles induces a beneficial alloying effect that facilitates uniform and dendrite-free plating of metallic Li (Fig. 17d). Thus, when combined with LPSC electrolyte, it endows Ah-class pouch cells a high energy density (> 900 Wh L^−1^) and superior cycling life of > 1000 times.

### External Field Regulation

**Fig. 18:**
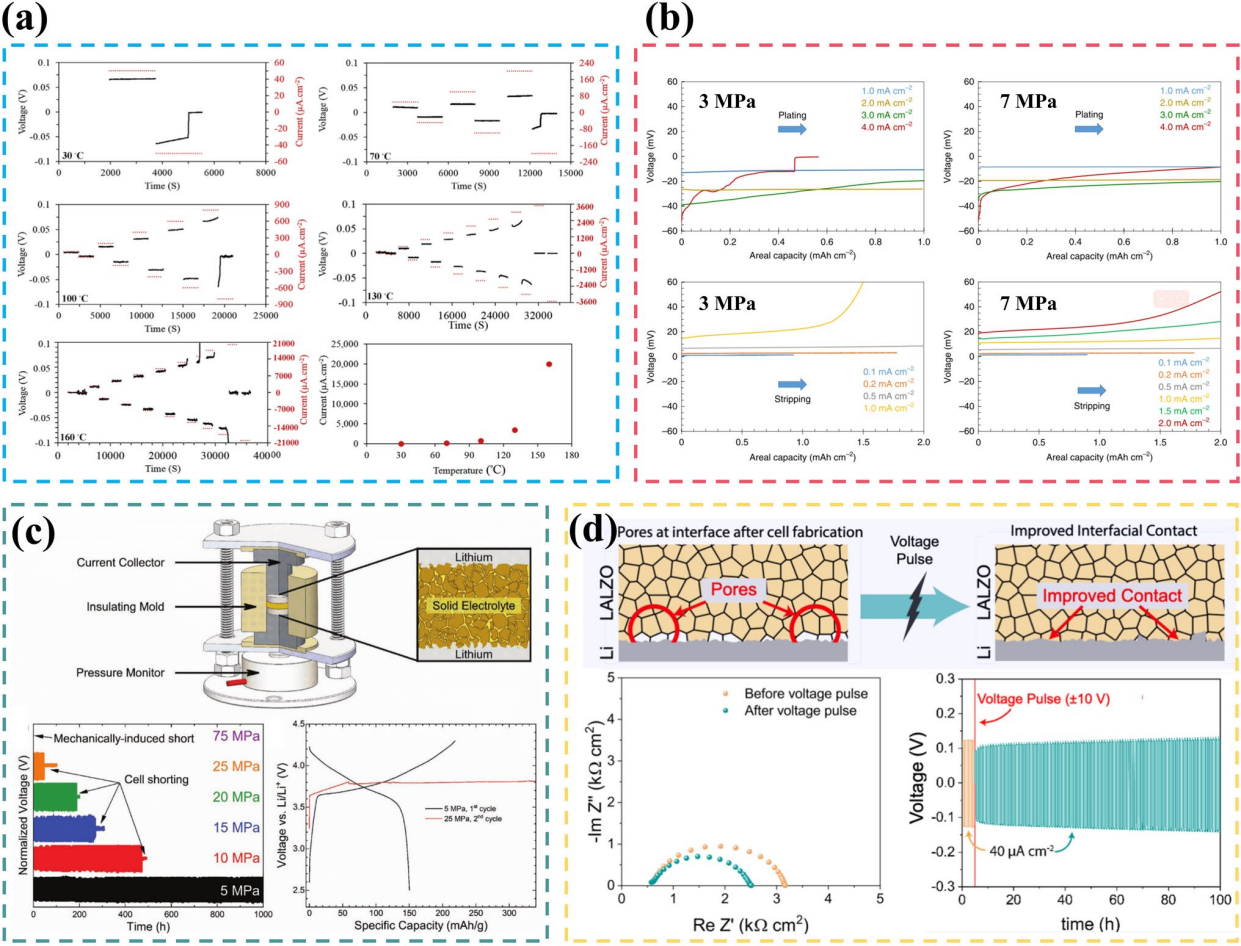
External field regulation for LMA in ASSLMBs. **a** CCD tests for Li/LLZO/Li cells at 30, 70, 100, 130 and 160 °C. Reproduced with permission from [390] Copyright 2016, Elsevier. **b** Voltage profiles for Li/LPSC/Li cells at different pressures and current densities. Reproduced with permission from [391] Copyright 2019, The Authors, published by Springer Nature. **c** Design of pressure control device as well as electrochemical performance for symmetric Li/Li cells and full cells under various external pressure. Reproduced with permission from [392] Copyright 2019, Wiley–VCH. **d** Schematic illustration of the effect of voltage pulse on stabilizing interface as well as the EIS spectra and polarization curves for symmetric Li/Li cells. Reproduced with permission from [395] Copyright 2022, American Chemical Society

On account of the nonvolatile and lower chemical reactivity of SSEs, ASSLMBs are suitable for high-temperature operation. Consequently, temperature exerts varying degrees of influence on both SSEs and LMAs [388, 389]. On one hand, it promotes Li^+^ transport through SSEs; on the other hand, as the temperature gradually approaches the melting point of Li (180 °C), it significantly enhances interface contact and kinetic properties [287]. Furthermore, at elevated temperatures, changes in metallic Li’s creep performance facilitate easier replenishment of stripped Li at the interface. As investigated by Sharafi et al., the resistance of Li-LLZO interface demonstrates a significant reduction to only 2.7 Ω cm^2^ upon heating to 175 °C [390]. As a result, this leads to increased CCD values of 0.05, 0.2, 0.8, 3.5 and 20 mA cm^−2^ corresponding to temperatures of 30, 70, 100, 130, and 160 °C (Fig. 18a).

The impact of external pressure on ASSLMBs is intricate. Kasemchainan et al. have concluded that the creep induced by pressure dominates the transportation of Li to the interface, rather than the process of Li diffusion [391]. When the stripping current density exceeds the replenishment rate of Li at the interface, voids will form and accumulate during cycling. This leads to an increase of local current density at the interface, ultimately evoking Li dendrite growth during plating, which further causes short circuits and cell failure. Comparing the voltage profiles during plating and stripping processes in Fig. 18b, it is observed that higher external pressure of 7 MPa, which significantly exceeds the yield strength of Li at 0.81 MPa, is conducive to reducing polarization and suppressing Li dendrites. However, excessive stress may facilitate the penetration of Li dendrites into SSEs. Meng’s research group has discovered that as stress gradually increases from 5 to 75 MPa (Fig. 18c), symmetric Li cells using LPSC electrolyte experience a significant decrease in lifespan and become more susceptible to spreading of Li dendrites [392]. Additionally, it leads to severe short-circuit issues during the 2nd cycle of assembled full cells when subjected to 25 MPa.

In liquid electrolytes, the pause interval of pulse current allows sufficient time for Li^+^ to diffuse from regions of high concentrations towards those with lower concentrations, resulting in a uniform Li deposition [393]. In the context SSEs, Reisecker et al. have proposed that the Li^+^ ion activity near the Li filament tip is critical for reliable cell cycling [394]. Limited by the geometric containment of SSEs, the practical current density at the crack tip can easily exceed the exchange current density, leading to lattice expansion and accumulated elastic energy that induces fracture in the surrounding area. While this critical activity requires a certain period to build up, it enables shorter duration current pulses to enhance both SSE/Li interface and cycling performance of ASSLMBs (Fig. 18d). It has been demonstrated that applying 1 MHz-pulsed currents can increase CCD of LLZTO pellets by a factor of six, as high as 6.5 mA cm^−2^. The presence of voids at the SSE/Li interface reduces the electrochemically active area and causes local current concentration. Parejiya et al. have demonstrated that pulse current near interfacial pores can improve interface contact through Joule heating [395]. The resulting local high temperature of LMA essentially implies a higher creep, which aid in reduced interfacial resistance. Moreover, the self-heating effect, which typically operates at a current density above 9 mA cm^−2^, can effectively eliminate Li dendrites and promote surface diffusion of Li^+^ ions to heal the dendritic LMA surfaces [52].

## Summary and Perspectives

**Fig. 19:**
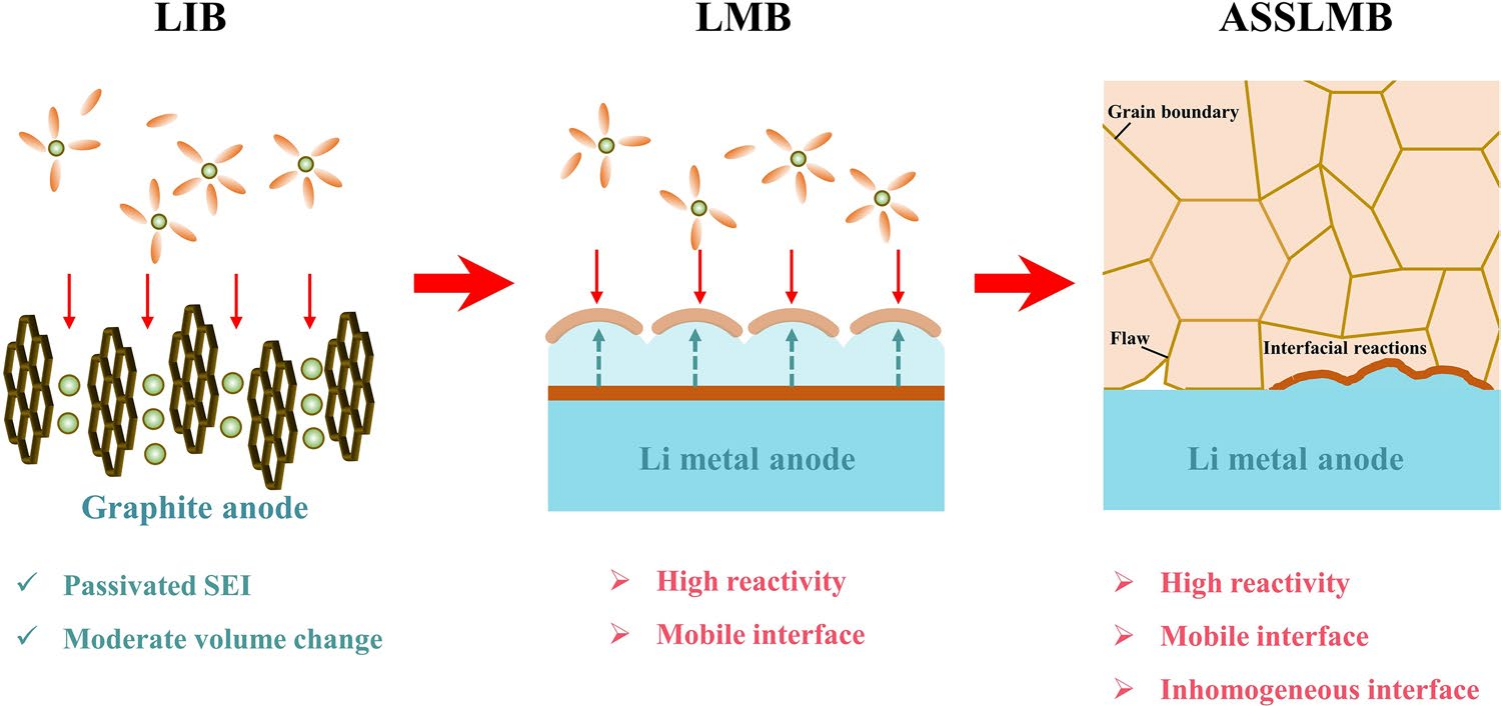
Summary of fundamental issues for the transition from LIBs to LMBs and ASSLMBs

From the perspective of enhancing energy density and reducing costs, the utilization of LMA represents one of the most promising developmental pathways for lithium batteries. Furthermore, to address the issues pertaining to high chemical reactivity and insecurity associated with LMA, its coordination with solid electrolytes is deemed imperative. Figure 19 summarizes the fundamental challenges in transition from commercial LIBs to LMBs and ASSLMBs. In LIBs, EC readily passivates the graphite anode, forming a stable SEI layer that reduces its electrochemical reactivity. Simultaneously, Li can efficiently intercalate between the graphite layers, merely resulting in moderate volume changes during cycling. However, in LMBs, metallic Li deposited is exposed to direct contact with organic electrolytes which may lead to active Li loss easily. Due to the lack of support from the host matrix, significant volume changes are experienced by LMA during cycling. The front edge of its electrochemical reaction constantly shifts with the amount of Li deposition, while the interface undergoes continuous migration and stress concentration that leads to SEI’s continuous destruction and reconstruction. This poses challenges in maintaining a stable electrochemical state. When applying the LMA to ASSLMBs, the properties of SSEs also significantly impact battery performance. However, high reactivity and migrated interfaces during Li plating/stripping processes present additional difficulties. Specifically, the inhomogeneity of SSEs, including defects, grain boundaries, the second phase, and insufficient interface contact can result in significant variations in electrochemical reaction degrees at the interface. Considering the non-fluidity and incompressibility of SSEs, such varied electrochemical states at the interface will further exacerbate the interfacial stress state, resulting in Li dendrite penetration and battery failure. Considering that SSEs lack fluidity and self-healing capabilities through diffusion like liquid electrolytes do, these uneven interface states will persist and worsen during subsequent cycles.

We have also summarized the enhanced strategies for LMB, which include interface engineering, 3D current collector design, electrolyte optimization, separator modification, alloyed anodes, and external field regulation. These strategies may exhibit slight differences in ASSLMBs, wherein separator modification is deemed necessary. The current perspective on research and development for transitioning from LMBs to ASSLMBs encompasses the following aspects:

1. The successful implementation of LMA necessitates the consideration of both chemical and physical interfaces, as well as their coupled effects. Therefore, it requires the integration of multiple improved strategies rather than relying solely on a single approach. Under the current state of research, significant progress has already been made in the development of liquid electrolyte-based LMBs. However, there still exists a substantial gap between these batteries and LIBs in terms of exceptional stability and cycling life. They are currently best suited for limited scenarios that demand high energy density while tolerating a limited lifespan.
2. It cannot disregard external factors such as temperature, thermal effects, charging/discharging procedure, magnetic fields, etc., which have significant impact on electrochemical performance of LMBs. A thorough understanding of the correlation between these factors and the transport process of Li^+^ along with Li deposition behavior is crucial for comprehending the electrochemical mechanism and potential failure modes of lithium batteries.
3. The propagation of Li dendrites remains the primary failure mode for LMBs, which underneath involves a complex mechanical-electrochemical coupling process, particularly in ASSLMBs. However, these challenges related to high chemical reactivity and non-uniform interface properties have yet to be fundamentally addressed, where the current advancement remains in the temporal delay of dendritic penetration. Further research is necessary to gain a deeper understanding of its intricate mechanisms. This also hinges upon the advancement of characterization techniques for lithium metal and solid electrolytes, enabling the exploration of uncharted microscopic phenomena.
4. Due to the inherent conflict between electrochemical stability and ionic conductivity, it is a formidable challenge for a single solid electrolyte to simultaneously fulfill all aspects of electrolyte property requirements. Consequently, an organic–inorganic composite electrolyte or semi-solid electrolyte containing some liquid components emerges as the optimal choice for striking a delicate balance between performance and safety.
5. Through the investigation of a wide range of LMBs spanning from liquid- to solid-state electrolytes, it has revealed the SEI formation mechanism, as well as the dissolution and solvation processes, thereby deepening our comprehension of interfacial electrochemical phenomena. These findings significantly contribute to our understanding of interfacial electrochemical phenomena, which in turn can be leveraged to enhance current LIB systems by minimizing electrolyte and active lithium losses, while simultaneously improving reactivity of active materials and overall battery safety.
